# Integrating metacognitive mechanisms optimizes EEG generative models via hierarchical regularization

**DOI:** 10.1016/j.isci.2026.115785

**Published:** 2026-04-16

**Authors:** Miaomiao Yu, Te Guo, Shangen Han, Na Xue, Wanying Yang, Junda Huang, Hongyu Chen, Cheng He, Jinhong Ding, Likun Xia

**Affiliations:** 1 Laboratory of Neural Computing and Intelligent Perception, College of Information Engineering, Capital Normal University, Beijing 100048, China; 2 School of Psychology, Capital Normal University, Beijing 100048, China; 3 Beijing University of Chemical Technology, Beijing 100029, China

**Keywords:** health sciences, medicine, medical specialty, electrodiagnostic medicine

## Abstract

Obtaining sufficient electroencephalography (EEG) signals for training deep neural networks (DNNs) in brain-computer interfaces (BCIs) is challenging due to individual differences in neural activity, which require large per-participant data to map signals to actions, while factors like movement artifacts often limit data collection. Existing advances mainly leverage generative models with various regularizers to produce sufficient EEG signals. However, selecting appropriate regularizers remains challenging. Inspired by metacognition, the human cognitive process that monitors and regulates learning and decision-making, we propose a metacognitive regulation module including three regularizers that explicitly capture EEG temporal dynamics and functional resolution, thereby improving both the diversity and similarity of generated data. Through extensive theoretical and empirical validation on two datasets, we demonstrate that our module: (1) significantly improves generative models for generating highly complex, realistic EEG activity; (2) improves generalization across different generative models; and (3) endows DNN models with enhanced human-like decision-making and adaptation capabilities.

## Highlights

• Proposes a metacognitive regulation module (MRM), simulating human metacognitive processes
• Balances similarity and diversity in generated EEG via temporal, spectral, and latent regularizers
• Achieves accuracy improvements across two datasets by leveraging the generated EEG signals

## Graphical abstract

**Figure:**
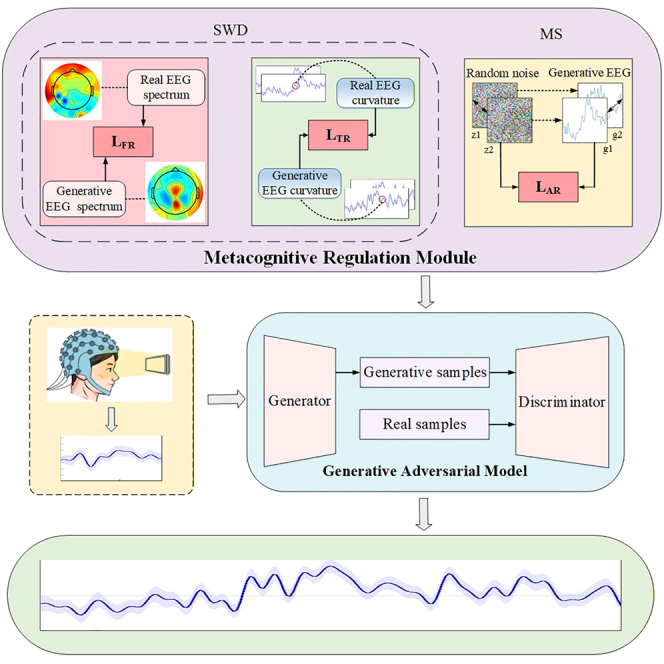

## Introduction

In recent years, deep learning models have become a mainstream method for electroencephalography (EEG) signal analysis due to their automatic feature extraction and end-to-end learning capabilities. The accuracy (Acc) and effectiveness of such models usually depend on the number of subjects (quantity) and the quality of the signal collected. Unfortunately, the acquisition of EEG signals faces critical uncertainties in both quantity and quality, including: (1) fluctuating subject states and individual differences lead to high variability in EEG signals and limited model generalization capability; some researchers^1^ integrate conditional identification information and leverage the interaction between EEG signals and individual attributes to enhance the model’s internal representation and improve decoding Acc. Leng et al.^2^ focused on the 20–40 Hz frequency band for EEG-based pain classification. Their method alleviates the impact of individual differences by capturing specific features from known subjects. (2) Unexpected personal/natural factors (e.g., emergencies and epidemics) may cause disruptions in data collection, reducing data usability and experimental efficiency. These challenges hinder researchers from rapidly obtaining sufficient and accurate data, ultimately affecting modeling Acc and effectiveness; (3) more importantly, in brain-computer interface (BCI) research, most studies focus on extracting features from existing EEG signals and mapping them to certain behaviors or functions for controlling machines. However, it remains questionable whether the mapping relationship can work in reverse, that is, whether it is possible to reconstruct (generate) EEG signals by integrating corresponding features, to validate the Acc and rationality of the feature extraction process, and effectively decode human cognitive behavior.

To address the issues above, researchers have attempted to apply generative models for EEG signal enlargement or generation, for example, generative adversarial networks (GANs)^3^^,^^4^ and related derivative models, such as EEG-GAN,^5^ recurrent GAN (RGAN),^6^ and conditional GAN (cGAN),^7^ and cc-LSTM-GAN.^8^ Diffusion-based generative models, such as probabilistic diffusion models^9^^,^^10^ and the coupled diffusion probabilistic model (D3VAE),^11^ have also found applications in this domain. While both GANs and diffusion models are generative frameworks capable of realizing EEG signal generation through the mapping from latent space to data space, their core mechanisms exhibit fundamentally difference. GANs operate within an adversarial game framework, wherein the generator and discriminator engage in a dynamic competition to directly approximate the target distribution. In contrast, diffusion models, inspired by physical principles of progressive generation, reconstruct the data distribution incrementally through forward noising and reverse denoising processes governed by Markov chains. Specifically, GANs offer the advantage of a single-step generation process, ensuring high computational efficiency. However, their limitations are pronounced because of the min-max strategy inherent in the original objective; the training process may diverge at any point due to instability, and the generated data may lose diversity due to mode collapse. On the other hand, diffusion models boast a relatively stable training process, enabling the gradual learning of complex data structures. Nevertheless, their multi-step iterative generation process requires a substantial quantity of high-quality training data. In scenarios with limited EEG data, diffusion models are susceptible to overfitting to noise patterns or artifact features, thereby compromising the quality of the generated data.

When selecting a generative model for EEG signals, it is imperative to balance computational efficiency, training stability, and data quality. Despite facing challenges such as mode collapse, overfitting leading to poor sample similarity, and a lack of diversity, GANs’ single-step generation and high efficiency make them particularly attractive for real-time applications with limited EEG resources.

To address issues of overfitting and poor signal quality, the introduction of regularization has become a fundamental strategy in model design. By constraining the learning process, regularization prevents models from memorizing noise in the training data and instead encourages the extraction of generalizable patterns, aimed at enhancing the similarity and diversity of generated signals.

For example, Wen et al.^8^ incorporated L2 regularization into a cc-LSTM-GAN to reduce overfitting. Their approach enables rapid domain adaptation by fine-tuning the generator using a small set of real EEG signals from new sessions or subjects. To preserve the statistical structure of the source data, they further introduced an inner-product regularizer—defined as the negative dot product between real and generated signals—into the generator’s objective function. Similarly, Huang et al.^12^ enhanced the diversity of generated data by optimizing an RpGAN with a relativistic alignment mechanism. To mitigate potential non-convergence, they added R1 and R2 regularizers, which impose zero-centered gradient penalties on the discriminator’s gradients for real and generated data, respectively, thereby smoothing the loss landscape and stabilizing training.

Existing research on regularization in EEG signal processing often adopts a results-driven methodology, selecting regularizers based on empirical outcomes rather than grounding them in the intrinsic mechanisms of neurophysiological signal generation or the theoretical properties of the regularizers themselves. This ad-hoc design paradigm restricts the targeted effectiveness and interpretability of the resulting computational models, limiting their ability to systematically address core challenges such as overfitting, limited decision-making quality, and poor cross-task transferability.

In a notable effort to bridge this gap between data-driven applications and neurophysiological grounding, Vahid et al.^13^ employed a conditional generative adversarial network (cGAN) for EEG generation, explicitly anchoring their work in the relationship between antagonistic cognitive functions (such as response execution and inhibition) and underlying neural principles. They introduced an L1 norm penalty between real and generated data as a regularizer for the discriminator, which was shown to improve the similarity of generated signals. This approach marks progress by connecting model design to a cognitive neuroscience framework.

However, their analysis did not extend to a principled exploration of the specific mechanistic action of the L1 regularizer within the adversarial training framework or a rigorous theoretical justification for its suitability over other potential regularizers for the EEG generation task. Consequently, critical aspects such as the enhancement of output diversity and the comprehensive mitigation of overfitting remain underexplored.^14^ Despite these advances, the field continues to grapple with fundamental issues where a more theoretically informed selection and analysis of regularizers could yield significant improvements in model robustness and generalizability.^15^

Current approaches to EEG generation, including the cGAN framework by Vahid et al.,^13^ often select regularizers based on empirical outcomes rather than a deep theoretical grounding. This results-driven methodology creates two fundamental shortcomings that hinder progress toward robust, human-aligned models. First, there is a lack of a principled representational framework that explicitly connects the mathematical function of a regularizer (e.g., an L1 penalty) to a defined cognitive or metacognitive principle. Consequently, these models are limited to promoting superficial signal similarity without capturing the underlying regulatory dynamics of the brain.^16^ Second, a fundamental architectural mismatch exists between the static, non-hierarchical design of common deep learning models and the dynamic, nested mechanisms of cognitive control observed in neuroscience. For instance, research shows that higher-order cognitive control, which governs the timing of rule application, is mediated by distinct neural oscillations (delta band) compared to the control of specific stimulus-action mappings (theta band).^17^ Standard discriminators or generators lack the structural analogs to model such hierarchical and regulatory processes, which are central to metacognition. This mismatch prevents models from generating signals with the rich, context-dependent variability characteristic of true neural processing.

We argue these shortcomings stem from a deeper issue: The field’s predominant focus on signal generation rather than cognitive modeling. To bridge this gap, we propose a paradigm shift grounded in metacognition—the psychological framework for the self-monitoring and control of one’s own cognitive processes.^18^ Cognitive neuroscience provides a basis for this approach, identifying specific neural correlates.^19^ For example, the precuneus is a key source for EEG activity (the P3 component) linked to metacognitive experiences.^20^ Furthermore, the prefrontal cortex, especially its most anterior frontopolar region, undergoes significant development in humans and is critically involved in the abstraction and integration necessary for metacognitive control.^21^

Integrating this perspective means moving beyond treating EEG as a mere signal to be reconstructed. Instead, we must model the generative process itself as a metacognitively guided one. A metacognitive system within a generative model would implement functions such as planning (setting generative goals), monitoring (assessing the quality of ongoing generation against internal standards), and evaluation (adjusting parameters based on outcome).^22^^,^^23^ This aligns with evidence that cognitive processes can be shifted toward a more controlled, “persistence-heavy” style through explicit instruction, which is reflected in more adult-like neural patterns.^18^

We therefore hypothesize that the regulatory function of a regularizer in a GAN operates analogously to human metacognitive control. This postulated equivalence is grounded in a shared principle of top-down regulation: Both systems implement high-level, goal-directed processes to monitor, evaluate, and adjust the operations of subordinate components (i.e., cognitive processes or network layers) to optimize overall performance and stability.^24^^,^^25^ To test this hypothesis, we design a metacognitive regulation module (MRM). The MRM architecturally emulates the metacognitive processes—such as planning, monitoring, and evaluation—of a human learner engaged in complex task performance. By embedding this regulatory intelligence, the MRM is engineered to enhance the cross-task generalization and decision-making quality of generative models. It integrates three domain-specific regularizers: A latent-space regularizer for manifold structure, a frequency-domain regularizer for spectral signatures, and a time-domain regularizer for temporal dynamics. Acting collectively, this multi-domain regulatory suite steers the adversarial training process toward a more stable and generalizable Nash equilibrium. We posit that by enforcing these neurobiologically and cognitively inspired constraints, the MRM will substantially improve the similarity of complex EEG pattern generation, leading to synthesized signals that exhibit enhanced diversity (exploring the valid space of neural patterns) and similarity (faithfully reproducing target signal characteristics).

**Figure 1:**
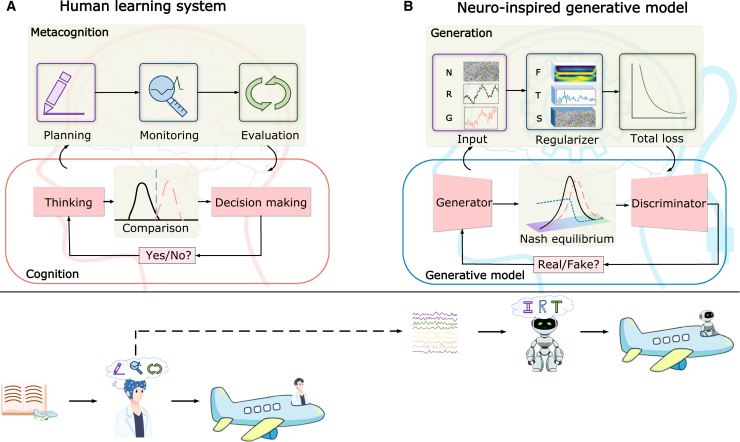
Neuro-inspired generative model with reference to the human learning system (A) Human learning system. Metacognition is the self-regulation within the human process of thinking to decision-making, which plays an important role in improving cognitive ability. It includes, for example, planning, monitoring, and evaluation of learning, thinking, and decision-making. (B) Inspired by such a human cognitive strategy, we proposed a metacognitive regulation module, which monitors and controls the training process of the generative model to enhance the decision-making capabilities of the model. The module integrates three regularizers: frequency domain regularizer, time domain regularizer, and the spatial regularizer, collectively driving the model to achieve Nash equilibrium.

We integrate the MRM into several established GAN-based EEG generation architectures and validate its performance on two distinct experimental datasets. Our results confirm that models equipped with the MRM demonstrate markedly improved cross-task adaptability and decision-making capability compared to their baseline counterparts. Figure 1 illustrates this core conceptual inspiration, drawing a direct analogy between the top-down regulatory loops in the human learning system (Figure 1A) and the integrative feedback mechanism of our proposed generative model (Figure 1B).

This work makes the following contributions.

• First, we establish a theoretical bridge between regularization in generative models and metacognition in human cognition, applying this principle to model design and grounding it in established experimental paradigms.
• Second, we introduce a MRM (MRM) that enables generative models to more accurately capture the temporal dynamics and functional resolution of EEG signals, thereby simultaneously enhancing the diversity and similarity of generated outputs.
• Third, our comprehensive evaluation framework, which extends beyond standard metrics to include multi-level qualitative visualization and a novel downstream validation task assessing the utility of mixed real-generated signals, offers a robust assessment of model performance.
• Finally, through extensive experiments, we demonstrate that models integrated with the MRM consistently outperform state-of-the-art methods across multiple quantitative and qualitative benchmarks.

## Results

### Experimental setup and EEG preprocessing

The development and validation of the MRM are meticulously crafted to account for the intricate temporal dynamics and the nuanced cognitive resolution of EEG signals. Additionally, it underscores the pivotal role that specific brain regions play in the efficacy of the model’s generation process. To encapsulate the diverse complexities of brain activity, we have meticulously chosen two datasets with varying levels of cognitive challenge: Bi2015a and PSFM. The Bi2015a dataset is adept at actively evoking the P300 response and allows for the modulation of the generated EEG amplitude in accordance with reward levels. Conversely, the PSFM dataset utilizes passively induced EEG signals that elicit P300 responses without conscious awareness. It is particularly significant that the former dataset requires a higher level of conscious control and reflects the brain’s rapid temporal responses. Our objective is for our generative models to swiftly and precisely mimic these spontaneously occurring EEG signals. The PSFM dataset, on the other hand, elicits subtle shifts in brain activity during decision-making, triggered by diverse stimuli such as cues or ambiguous prompts, thereby showcasing a robust resolution of cognitive functions. We aspire for our generative models to be sensitive enough to discern these minute variations and to generate EEG signals that can effectively differentiate between these nuanced changes.

To obtain satisfactory computational efficiency, the sampling rate of the Bi2015a dataset was chosen to be 256 Hz, while the PSFM dataset maintained a sampling rate of 500 Hz. During the preprocessing stage, to remove the power-line noise, the signals from these datasets underwent bandpass filtering within the range of 0.01 Hz–40 Hz, utilizing a finite impulse response (FIR) filter. We focused on specific signal channels, including five channels located in the parietal cortex region that are implicated in perceptual decision-making processes. Subsequently, the filtered data were standardized using the MaxAbsScaler method to guarantee that the signal amplitudes were optimally suited for model training purposes. Moreover, we complemented this automated processing with a manual selection procedure to refine the synthetic EEG signals based on the characteristic features of genuine EEG activity. This involved a screening process of the generated EEG signals, starting from the later epochs of the training phase and proceeding in batches. The selection criterion was guided by the distinctive activity patterns of real EEG signals, such as the P300, to ensure the inclusion of only the high-quality generated EEG signals in our dataset.

### Correlation of the metacognition mechanism and the generative model

GANs, comprising a generator and a discriminator, are generative models that draw inspiration from game theory. Their core concept is based on a min-max game played between two competing neural networks, with the objective of learning the distribution of target data. During the training phase, the generator network ingests random noise vectors, typically drawn from simple prior distributions such as the standard normal or uniform distribution, and endeavors to generate data that are indistinguishable from real data. Concurrently, the discriminator network is responsible for evaluating both real and generated samples, striving to accurately classify them as either real or fake. This adversarial training process drives both networks to evolve iteratively, ultimately reaching a Nash equilibrium where the generator becomes adept at producing increasingly generated data.

In the realm of computer vision, GANs have demonstrated the remarkable ability to generate high-resolution images that are virtually indistinguishable from real images. Unfortunately, when it comes to replicating the intricate physiological characteristics of EEG signals, the EEG-specific GANs currently in use are inadequate in generating signals that truly reflect the nuances of real EEG signals. The generated EEG signals still encounter substantial challenges, particularly in achieving a high degree of similarity and diversity to real EEG signals.

The reasons for this phenomenon are multifaceted. From a signal processing perspective, one possible reason is that GAN was originally designed for the gaming industry and later transitioned to image generation. Since there are similarities in the visual aspects of gaming and image processing, the design of GAN is naturally suited for image processing. However, when applying GAN to time-series signal generation, especially EEG signals, it is necessary to understand the fundamental differences between EEG signals and images. Unlike image generation using CNNs in GANs, EEG signal generation may result in the incorrect reproduction of the spectrum distribution in real signals, leading to high similarity. In addition, existing GAN-related models struggle to fully capture the temporal dynamics of EEG signals due to subtle variations, resulting in poor diversity of generated signals and causing overfitting.

One effective approach is to add regulation term to minimize the difference between training and testing. However, it is found that the model still suffers from insufficient generalization ability during the generation process. One possible reason is the lack of a theoretical foundation to support the selection of these regularization terms. Previous studies primarily approached the choice of regularization terms from a result-oriented perspective, rather than designing the generative model by integrating the underlying mechanism of the generation process with the characteristics of the regularization terms. This lack of targeted design has led to the persistence of the aforementioned issues.

Beyond technical reasons, there is a deeper rooted in the cognitive perspective. EEG signals contain richer and more complex information that deeply reflects human thinking and cognitive processes, whereas images are more straightforward in presenting the intuitive content of objects. Therefore, GANs originally designed for image processing are not directly applicable to the analysis of EEG signals, which are inherently more complex. To address this challenge, some researchers have specifically designed generator and discriminator networks for time-series signals. For example, the cc-LSTM GAN model proposed by Wen et al.^8^ replaces traditional convolutional layers with LSTM layers to better capture the long-term dependencies in time-series data. The WaveGAN model designed by Donahue et al.^26^ introduces one-dimensional convolutional kernels and effectively captures the short-term dependencies of the signal using the local receptive field in the time domain, thus adapting to the one-dimensional nature of time-series data. In contrast, Vahid et al.^13^ designed the generation process from a cognitive perspective by carefully selecting the Go/Nogo task as their research subject, where the two conditions exhibit significant antagonism in cognitive functions. This design provided the cGAN with a more diverse training dataset, thereby significantly enhancing model performance. However, they failed to integrate the intrinsic mechanisms of the model with cognitive functions to further optimize the model design.

Inspired by this study and recognizing the importance of time-series signals in the EEG generation framework, we propose a hypothesis that regularizers function similarly to metacognition in cognitive processes. In the overall model architecture, input noise, real samples, and generated samples collectively form the planning component of metacognition, as they jointly define the objectives and initial conditions of the generation task. Regularizers simulate the monitoring function of metacognition by imposing constraints on the generation process, while the overall objective function serves as the evaluation component of metacognition, continuously optimizing the model and dynamically adjusting generation strategies to drive the model toward the Nash equilibrium.

**Figure 2:**
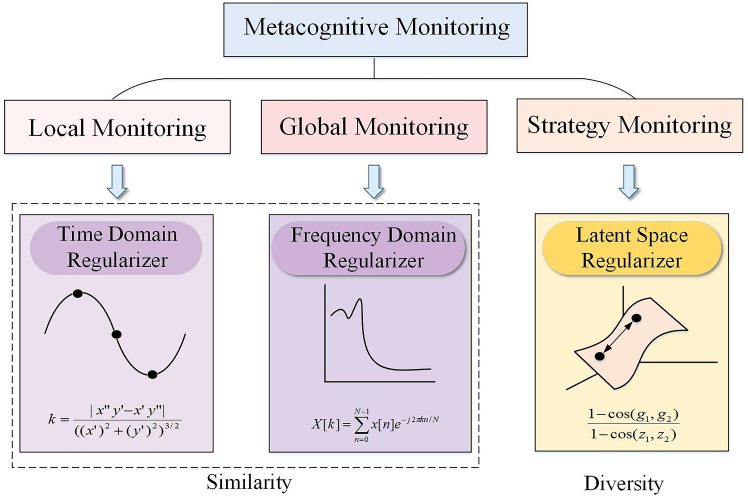
The selection of regularizers with reference to metacognitive monitoring

We further explored the metacognitive monitoring mechanism, which typically encompasses three operational dimensions: local monitoring, global monitoring, and strategy monitoring. Local monitoring refers to the short-term monitoring of an individual’s current learning process. Global monitoring refers to the long-term monitoring of an individual’s current learning process. Knowledge organization strategy monitoring pertains to the individual’s monitoring of the learning strategy currently being adopted. Inspired by these principles, as shown in Figure 2, we introduced three types of regularizers to model metacognitive monitoring functions: (1) Time domain regularizer (TR), which captures local waveform characteristics by computing the curvature of EEG signals and simulating local monitoring; (2) Frequency domain regularizer (FR), which extracts global spectral features of EEG signals via the Fourier transform, corresponding to global monitoring. These two regularizers aim to enhance the similarity between generated and real signals in the time and frequency domains. (3) Latent space regularizers, which measure the ratio of differences between input noise and generated vectors to promote diversity in the latent space, emulating the strategic adjustment function of strategy monitoring. Together, these three regularizers synergistically form the MRM, effectively modeling the monitoring functions of metacognition.

**Figure 3:**
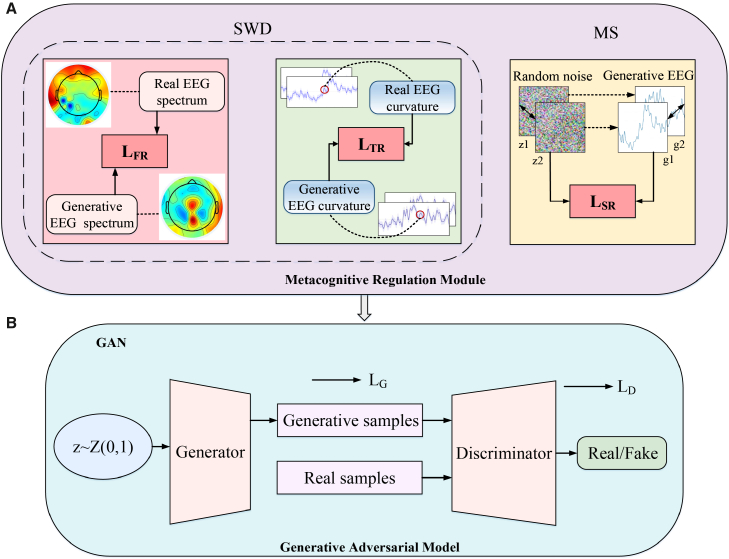
The framework of the proposed metacognitive regulation module that is integrated into the generative adversarial module (A) Metacognitive regulation module contains three regularizers: LFR, LTR, and LSR. LFR and LTR decrease the SWD metric, LSR is implemented to enhance the MS metric. Both z1 and z2 are random noise, and g1 and g2 are generated EEG signals. (B) Generative adversarial module consists of a number of distinct GANs: WaveGAN, WGAN-GP, and CWGAN-GP.

As shown in Figure 3, the overall framework of the generative model includes a MRM and a generative adversarial model. The MRM includes three regularizers, namely the FR, TR, and latent space domain regularizer (SR). Specifically, FR and TR respectively calculate the amplitude spectrum and curvature differences between real data and generated data, and generate the corresponding FR loss LFR and TR loss LTR. SR loss LSR is used to penalize the dissimilarity between two generated EEG signals and the dissimilarity ratio between their corresponding random noises in latent space. Notably, the module is integrated into the generator of the generative model instead of the discriminator. This is because it can help the generator better understand the current generation state and dynamically adjust generation strategies during the generation process. Whereas introducing the module into the discriminator may not provide a direct advantage, as the discriminator aims to perform classification between real and generated samples, rather than focusing on specific generation strategies.

To improve the stability of GANs and their applicability to EEG signals, we have selected three modified GAN models as the base models to validate the effectiveness of the MRM: WGAN-GP, CWGAN-GP, and WaveGAN. The experimental results presented in the following sections are based on WaveGAN as an example, while the results of other models are detailed in the “selection of hyperparameter” section. The metacognitive generative model refers to the improved model obtained by integrating the MRM into the WaveGAN framework. All results were averaged over 3 runs, with error bars representing the standard error of the mean.

### Ablation experiments

**Figure 4:**
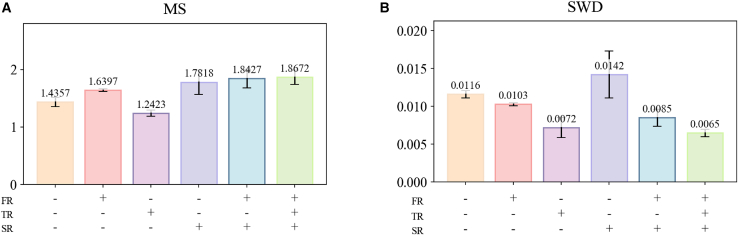
The ablation experiments for the metacognitive regulation module (A) The performance of the module in terms of the MS metric. (B) The performance of the module in terms of the SWD metric. Data are represented as mean ± SD.

The MRM contains three regularizers designed to enhance the similarity and diversity of the generated signals: FR, TR, and SR. To validate the impact of these regularizers on the performance of the generative model, an ablation study using the MS and SWD metrics was conducted using the Bi2015a dataset. The experimental results are presented in Figure 4. It is seen that, compared to the baseline model, introducing a single regularizer leads to the following: FR reduces the SWD value from 0.0116 to 0.0103, while TR lowers the SWD value to 0.0072. This demonstrates the effectiveness of FR and TR in improving signal similarity from the frequency and time domain perspectives, respectively. Simultaneously, the integration of SR has elevated the MS value from 1.4357 to 1.7818, signifying that SR effectively, indicating that SR effectively enhances signal diversity by penalizing discrepancies between noise and the generated signals. Moreover, the combination of FR and SR results in a 0.0609 increment in the MS value compared to using FR alone, and a 0.0018 decrement in the SWD value compared to using SR alone, indicating that the synergy of these two regularizers produces superior outcomes across the evaluated metrics. Building upon the effective performance improvement achieved by combining these two regularizers, TR is incorporated in this experiment to impose temporal constraints on the generated EEG signals, with the objective of further refining signal similarity. The experimental results show that the SWD value decreases to 0.0065, and the MS value increases to 1.8672, achieving optimal values for both similarity and diversity. These results collectively indicate that all three regularizers within the MRM contribute to improvements in both similarity and diversity metrics, thereby proving the effectiveness of the module.

### MRM evaluation across different generative models and datasets

To investigate the applicability of the MRM across different generative models, we integrate it into three distinct generative models: WaveGAN, WGAN-GP, and CWGAN-GP using the Bi2015a and PSFM datasets.

#### Quantitative analysis

**Figure 5:**
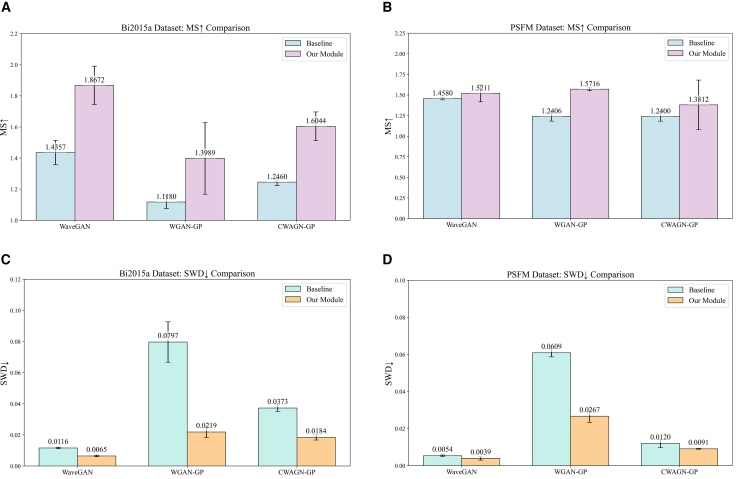
The quantitative evaluation for the metacognitive regulation module (A) Comparison results of Bi2015a on MS. (B) Comparison results of Bi2015a on SWD. (C) Comparison results of PSFM on MS. (D) Comparison results of PSFM on SWD. Data are represented as mean ± SD.

A quantitative analysis employing the MS and SWD metrics was conducted. As illustrated in Figure 5, the generative models enhanced with the MRM generally demonstrate significant improvements in both similarity and diversity across various datasets, thereby verifying the module’s effectiveness in optimizing the quality of generated samples.

Specifically, on the Bi2015a dataset, the MS value of the WaveGAN model increases from 1.4357 to 1.8672, indicating a substantial enhancement in the diversity of the generated samples. Concurrently, the WGAN-GP model exhibited the best performance in terms of similarity, with its SWD value decreasing by 0.0579, reflecting a significant improvement in the proximity between the generated samples and the real sample distribution. Among the three models, WaveGAN demonstrated superior performance on both metrics for the Bi2015a dataset, highlighting the effectiveness of its network architecture specifically designed for time series data. On the PSFM dataset, the WGAN-GP model shows the most significant improvement in diversity, with an MS value increase of 0.301. Meanwhile, the CWGAN-GP model has achieved the most notable improvement in similarity, with its SWD value decreasing from 0.0120 to 0.0091, demonstrating stronger simulation capabilities. When comparing the performance of the three models on the PSFM dataset, although the MS value of WaveGAN is slightly lower than that of WGAN-GP, its SWD value is significantly lower, rendering WaveGAN the overall best-performing model.

#### Qualitative analysis

**Figure 6:**
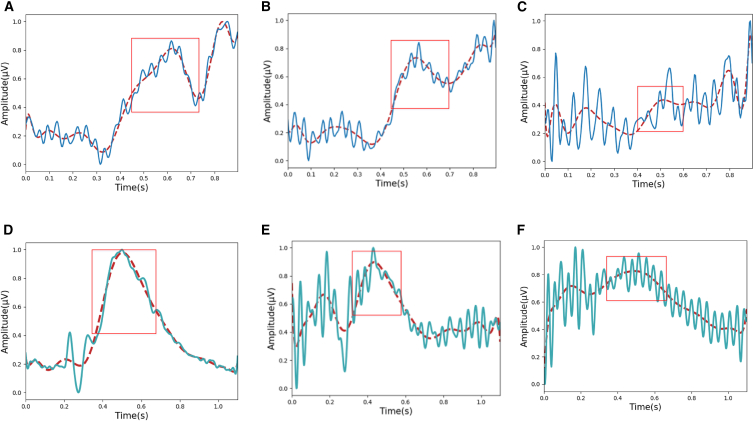
Comparison of real EEG and generated EEG signals from the F7 electrode in the Bi2015a (A–C) and PSFM datasets (D–F) (A) Display of Bi2015a signals. (B–C) Signals generated by MetacGAN and GAN on the Bi2015a dataset, respectively. (D) Display of the PSFM signals. (E–F) Signals generated by MetacGAN and GAN on the PSFM dataset, respectively. The solid line represents the EEG signal, while the dashed line corresponds to its fitted trend line.

In this section, we conduct a visual analysis by juxtaposing the temporal and spectral maps derived from the generated EEG signals with real ones. In examining the temporal maps, one can discern whether P300 is present in the generated signals, thereby determining the similarity between the generated and the real signals. Given that the amplitude of P300 is relatively small, it is difficult to directly identify the P300 potential generated by a single stimulus from complex EEG signals. To address this issue, we employed an averaging algorithm to eliminate the random noise in EEG signals. By aligning the signals in time before averaging them, with the increase in the number of repetitions, the background noise, due to its randomness, cancels out, while the P300 gradually emerges. This process significantly improves the signal-to-noise ratio, allowing the P300 to be clearly identified. To further enhance the quality of data preprocessing, we applied min-max normalization to the signals to ensure their comparability and consistency. To more clearly observe the P300 of EEG signals, we employed polynomial fitting to generate a trend line for the signal. Through multiple experiments, it was observed that a 15th-order polynomial trend line most effectively highlights the characteristics of the P300. Figure 6 show cases the visualization outcomes within the time domain. In general, the EEG signals produced by the metacognitive regulation model exhibit a striking resemblance to the real EEG signals, particularly in the manifestation of the P300.

In the Bi2015a dataset, a distinct contrast in voltage fluctuations around the 300 ms mark is evident when comparing the generated EEG signals by MetacGAN (Figure 6B) with those produced by GAN (Figure 6C). As shown in the red box, Figure 6B exhibits a more prominent positive peak in the P300, indicating that the MRM has enabled the GAN to better capture the characteristics of the P300 in real EEG signals. Moreover, the oscillation pattern of the EEG signal (Figure 6B) exhibits a closer affinity to the real EEG signals (Figure 6A) than the one in Figure 6C, demonstrating the module’s proficiency in encapsulating the intricate temporal dynamics of EEG signals within the Bi2015a dataset. In the PSFM dataset, although the generated signal is not as smooth as the real one, the red trend line clearly highlights the P300. The EEG signals generated by MetacGAN (Figure 6E) show a distinct positive peak in the 300 ms–500 ms voltage changes, as highlighted by the red boxes, unlike the signal generated by the GAN (Figure 6F). This indicates that MetacGAN is adept at capturing the P300 in real EEG signals. It is noteworthy that the generated EEG signals not only replicate the real EEG signals but also effectively address the issue of mode collapse that may occur due to gradient vanishing, the typical manifestation of which is the generation of repetitive simplified waveforms. Taking Figure 6E as an example, the EEG signals generated by MetacGAN retain the P300 of real signals, and their waveform changes were complex and diverse, whereas Figure 6F showed the emergence of numerous repetitive simple waveforms after approximately 500 ms, indicating that the generated signals by MetacGAN possess diversity. Furthermore, we evaluated the impact of the MRM on diffusion models, with the relevant results detailed in the “performance evaluation of the MRM in diffusion-TS models” section.

**Figure 7:**
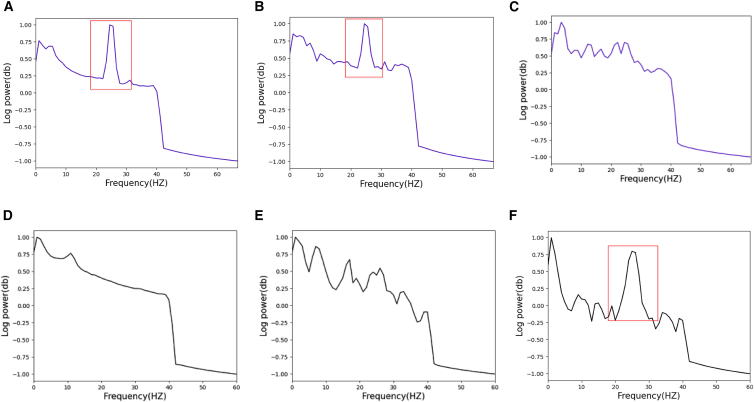
Comparison of PSD between real EEG and generated EEG signals from Bi2015a (A–C) and PSFM datasets (D–F) (A) Display of Bi2015a signals. (B and C) Present the signals generated by MetacGAN and GAN on the Bi2015a dataset, respectively. (D) Display of the PSFM signals. (E–F) Signals generated by MetacGAN and GAN on the PSFM dataset, respectively.

To further evaluate the quality of the generated signals, we investigated whether they exhibit power spectral density (PSD) similar to that of real signals. To assess this, we adopted the Welch PSD estimation method to analyze their power distribution across various frequency bands. This technique involves segmenting the signal into overlapping windows of a defined duration, applying the fast Fourier transform (FFT) to each segment, and then computing the mean power of the FFT coefficients across all overlapping windows. To remove the power-line noise, the frequency range was restricted to 0.01–40 Hz. To balance the trade-off between frequency resolution and temporal dynamics in EEG signals, we selected a 1-s window length. Longer windows yield higher frequency resolution but lower temporal resolution, potentially losing short-term dynamic features. Shorter windows enhance temporal resolution, better capturing short-term features, but reduce frequency resolution, making it challenging to analyze low-frequency components. Additionally, to enhance the robustness and smoothness of spectral estimation, we set the window overlap rate to 50%. This configuration increases the number of averaged windows, reduces the variance of the spectral estimate, and minimizes information loss at window boundaries. Figure 7 delineates the visualization of frequency domain results. Overall, compared to the GAN, the PSD of EEG signals generated by MetacGAN exhibits a higher degree of similarity to that of real signals. In the Bi2015a dataset, within the frequency band of 20 Hz–30 Hz, the real EEG signals (Figure 7A) manifest pronounced power peaks, as highlighted by the red boxes. The EEG signals generated by MetacGAN (Figure 7B) exhibit even more accentuated peak characteristics within the spectral range as compared to those produced by the base model (Figure 7C).

In the PSFM dataset, the PSD of real EEG signals (Figure 7D) exhibits a stable energy trend, whereas GAN-generated EEG signals (Figure 7F) show significant energy fluctuations in the 20–30 Hz frequency band. In contrast, although the MetacGAN-generated EEG signals (Figure 7E) are less smooth than the real signals, they display no pronounced energy spikes at the same band observed in the GAN, demonstrating the effectiveness of the MRM in spectral modulation.

In summary, compared to GAN, the EEG signals generated by MetacGAN are more similar to the real signals in terms of P300 and PSD, while also exhibiting notable diversity rather than merely replicating real signals. Unfortunately, there are still some differences between the generated and real signals. Therefore, it is necessary to assess whether the generated signals are qualitative enough to effectively assist various downstream applications, e.g., optimization of classifiers. Furthermore, we also evaluate the quality of generated EEG signals on WGAN-GP (MetacWGAN) and CWGAN-GP (MetacCWGAN) models with the embedded MRM, with detailed analyses provided in the “visual analysis through the juxtaposition of temporal and spectral maps” section.

### Classification performance of MRM-based models using different mixed ratios of EEG signals

We combine the generated EEG signals with real ones in different proportions to optimize various classification models. The aim is to obtain satisfactory classification results even with limited real signals, thereby verifying the quality of these generated signals and further evaluating the effectiveness of the MRM. For this purpose, we selected EEGNet^27^ and DsMuLBHiTA^28^ as our classification models, applying them to the Bi2015a and PSFM datasets. The rationale behind selecting these models lies in their optimized design for capturing the temporal characteristics of EEG signals. Specifically, EEGNet, a CNN-based model, effectively extracts the time-frequency features of EEG signals through depthwise separable convolution layers. In contrast, DsMuLBHiTA is constructed based on a BLSTM framework and incorporates an attention mechanism, endowing it with robust temporal modeling capabilities. This enables it to effectively capture the non-stationary characteristics and global dependencies in EEG signals. Moreover, our experimental design comprehensively covered both single-channel and five-channel data from the Bi2015a and PSFM datasets, and constructed two and three classification tasks, respectively. Through this diverse experimental setup, we could systematically evaluate the enhancement effects of the generated EEG signals under different data sources, channel configurations, and task complexities.

In this experiment, the dataset was divided into a training set and a test set in an 8:2 ratio. Subsequently, 5-fold cross-validation was applied to the training set to further optimize model performance. Specifically, the training set was evenly split into five subsets, with each iteration selecting four subsets as training data and the remaining one subset as validation data. Through iterative cycles, the model was trained on different training subsets and evaluated on the corresponding validation sets, thereby enhancing its stability and generalization ability. The classification model was implemented, built, and trained using the TensorFlow 2.0 framework and computed on an NVIDIA Quadro RTX 6000 graphics processing unit (GPU). To optimize network performance, batch normalization and dropout strategies were incorporated into the model. Additionally, categorical cross-entropy (CE) was selected as the loss function in the experiment to evaluate the model’s performance on both datasets.

**Figure 8:**
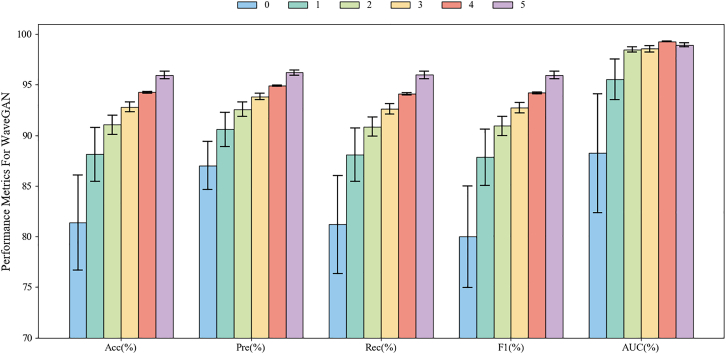
Two-class classification results based on different mixed ratios of single-channel EEG signals using EEGNet in the Bi2015a dataset. “0” denotes the results using only real EEG signals as training data, while “1, 2, 3, 4, 5” represent the results when the generated EEG signals are one to five times the amount of the real ones, respectively. Data are represented as mean ± SD

**Figure 9:**
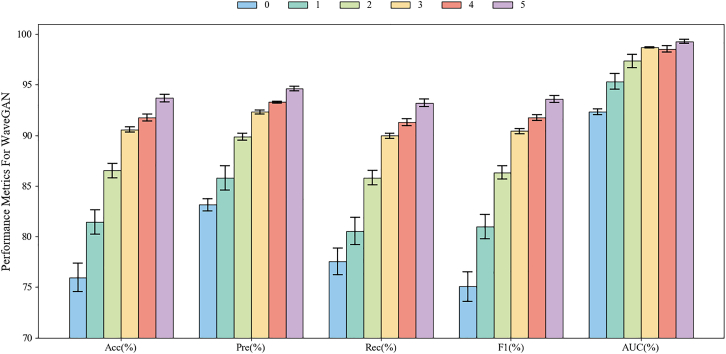
Three-class classification results based on different mixed ratios of single-channel EEG signals using EEGNet in the Bi2015a dataset. The baseline condition (“0”) utilizes only real EEG signals for training, while “1” through “5” indicate that the generated signals are augmented to 1–5 times the volume of the real signals, respectively. Data are represented as mean ± SD

**Figure 10:**
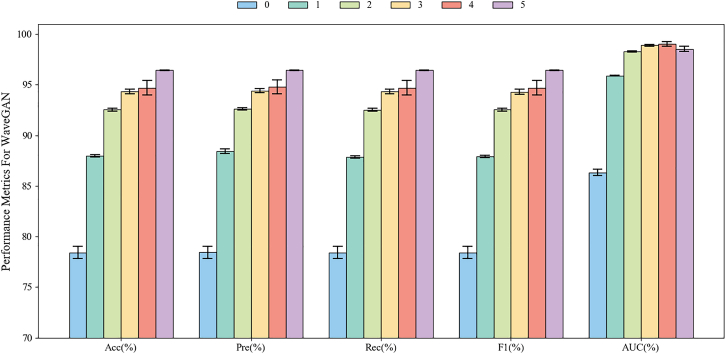
Two-class classification results based on different mixed ratios of five-channel EEG signals using DsMuLBHiTA in the PSFM dataset. “0” denotes the results using only real EEG signals as training data, while “1, 2, 3, 4, 5” represent the results when the generated EEG signals are one to five times the amount of the real ones, respectively. Data are represented as mean ± SD

**Figure 11:**
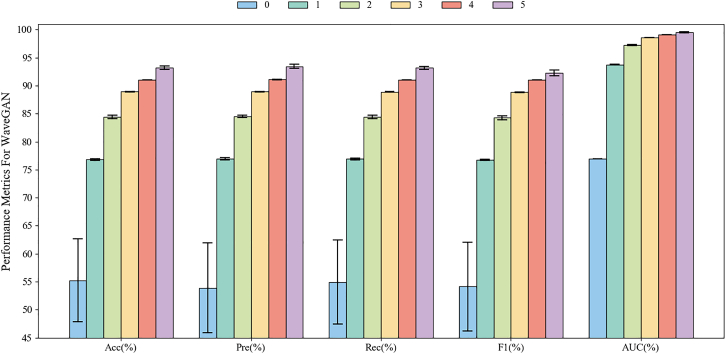
Three-class classification results based on different mixed ratios of five-channel EEG signals using DsMuLBHiT in the PSFM dataset. Ratios “0” (baseline) to “5” indicate the proportion of generated to real EEG signals used for training, with “0” representing real data only and “1–5” representing 1× to 5× augmentation of generated data. Data are represented as mean ± SD

The experimental results are illustrated in Figures 8, 9, 10, and 11, with the remaining results detailed in the “analysis of experiment classification results” section. In each experiment, the results from the mixed signals were compared and analyzed against those obtained using only real EEG signals. Here, “0” denotes the results using only real EEG signals as training data, i.e., baseline, while “1, 2, 3, 4, 5” represent the results when the generated EEG signals are one to five times the amount of the real ones, respectively. For example, “2” indicates a ratio of 2:1 between the generated signals and the real ones. The reason for limiting the proportion of generated signals to a maximum of five times is based on the empirical conclusions from relevant literature. Although the gradual introduction of high-quality generated signal generally helps to improve model performance, excessive quantities may lead to performance saturation or even decline, and detailed saturation analysis in “analysis of trends in model Acc improvement” section. This is because maintaining a distributional balance between real and generated signals is crucial while leveraging the advantages of generated data. If the proportion of generated data is too high, it may disrupt this balance, leading to model learning bias and ultimately resulting in suboptimal performance.^29^^,^^30^

Figure 8 presents a comparison of EEGNet’s two-class classification performance on the Bi2015a dataset using single-channel EEG signals. In general, the results of “5” outperform the other ratios across all the metrics. And for each metrics, there is an increase trend found as the ratio of real data to data generated by MetacGAN increases. Specifically, the incorporation of generated signals results in a 14.56% increase in classification Acc relative to the use of real signals alone, i.e., “0”. In terms of precision (Pre) and recall (Rec), improvements of 9.19% and 14.77% can be found, respectively, compared to the results from “0.” These results indicate that generated signals not only bolster the model’s ability to identify positive samples but also enhance its classification coverage. Both F1 scores (F1) and AUC exhibit consistent improvements with increasing proportions of generated signals, further corroborating the positive impact of generated signals on stability and generalization during training. Thus, these findings demonstrate that incorporating single-channel generated EEG signals significantly enhances model performance in binary classification tasks under the EEGNet.

We then conduct three-class classification experiments on single-channel EEG data from the Bi2015a dataset using EEGNet, and the results are illustrated in Figure 9 It is evident that the results of “5” demonstrate superior performance across all metrics compared to other ratios. Notably, the MetacGAN with “5” has achieved a remarkable 17.72% improvement of Acc relative to “1,” while maintaining consistently low SD across all generated signal proportions. Other metrics, including Pre, Rec, and AUC, also see improvement of 11.50%, 15.65%, and 6.96%, respectively, highlighting the enhanced discriminative ability and classification consistency. Importantly, the F1 exhibits a consistent upward trend with increasing proportions of generated signals and a significantly lower standard deviation than the baseline, further reinforcing the stability of the generated signals. These results indicate that incorporating single-channel generated EEG signals significantly enhances model performance in ternary classification tasks under the EEGNet framework, further validating the efficacy of the proposed module and three GANs in EEG signal augmentation. Compared to the binary classification results presented in Figure 8, the performance improvement observed in multi-class tasks is more substantial, underscoring the greater utility of generated EEG signals in complex classification scenarios.

We also perform two-class classification results of five-channel EEG on the PSFM dataset using the DsMuLBHiTA, the results are shown in Figure 10. It is found that the results of “5” outperform other ratios across all the metrics. For example, an improvement of 18.01% from Acc on “5” is achieved as compared to “0.” The similar results of Pre, Rec and F1 to Acc can also be found, implying the substantial enhancement in discriminative power and classification consistency. Notably, the SD from “4” across these metrics is slightly higher than “0,” possibly due to minor instabilities in handling large-scale generated data, leading to marginal performance fluctuations. In terms of AUC, the SD with higher proportions of generated signals is significantly lower than the baseline “0,” further validating the positive contribution of generated signals to model discrimination and stability. These results confirmed that incorporating five-channel generated EEG signals markedly improves model performance in binary classification tasks under the DsMuLBHiTA framework. Additionally, by comparing with the results using single-channel EEG signals in the “analysis of experiment classification results” section, we found that the performance improvement was more significant under multi-channel task conditions. For example, Acc with single-channel increased by 15.28% under “5” compared to the baseline “0,” while Acc with five-channel increased by 18.01%. This comparative result indicates the utility of the generated EEG signals in multi-channel task scenarios.

Subsequently, we validate the DsMuLBHiTA on the same dataset for multiclass classification. Figure 11 illustrates the three-class classification results. The metacGAN, augmented with 5-fold generated signals, achieved an exceptional Acc improvement of 37.97% compared to the baseline, utilizing only real signals. Notably, the SD remained consistently low across all generated signal proportions. Under the “5,” the Pre and Rec metrics improved by 37.54% and 38.22%, respectively, demonstrating significant enhancements in discriminative ability and classification consistency, with the SD of these metrics remaining below the baseline. Moreover, the F1 and AUC increased by 38.14% and 22.54%, respectively. It is particularly noteworthy that the SD of AUC under “4” was lower than “0,” while other proportions exhibit slightly higher but statistically insignificant deviations, demonstrating robust overall stability. These results strongly affirm that incorporating five-channel generated EEG signals substantially enhances model performance in ternary classification tasks under the DsMuLBHiTA, further validating the effectiveness and adaptability of the proposed module and associated GANs in EEG signal augmentation. A comparison with the results using single-channel EEG signals in the “analysis of experiment classification results” section shows that performance improvement is more significant under multi-channel task conditions. For example, the Acc with single-channel increased by 28.90%, while the Acc with five-channel increased by 37.97%. This comparative result was similar to the findings in Figure 10, indicating the utility of multi-channel generated EEG signals in different classification tasks.

### Visualization of generated EEG signals using t-SNE: A feature space analysis of classification models

**Figure 12:**
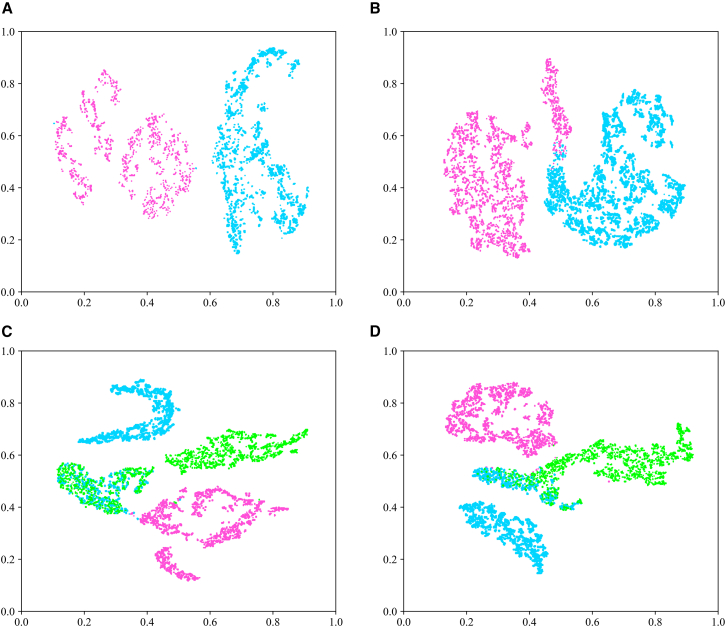
The t-SNE visualization provides an effective representation of the distribution of high-dimensional features extracted from the last layer of the EEGNet model in the Bi2015a dataset. (A)-(B). Illustrate the feature distribution domains for two-class classification tasks, comparing single-channel versus five-channel data configurations. (C)-(D). Depict the feature distribution domains for three-class classification tasks, again contrasting single-channel against five-channel data setups.

**Figure 13:**
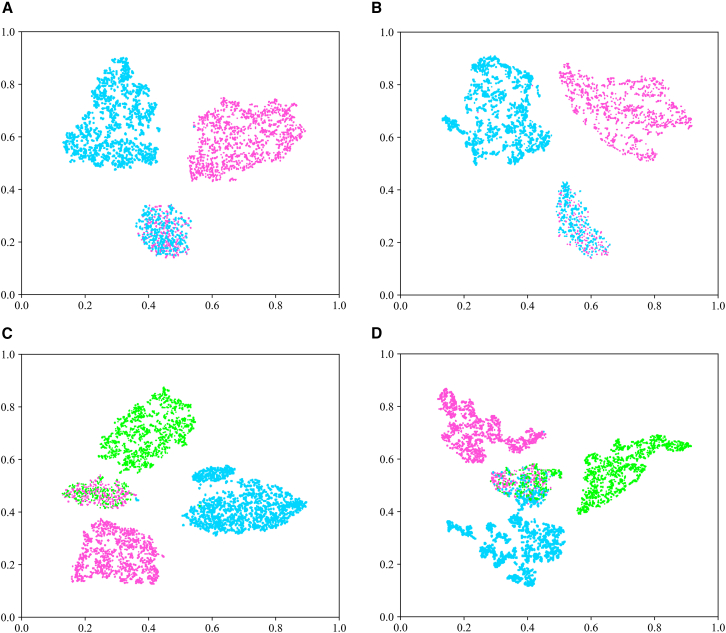
The t-SNE visualization effectively represents the distribution of high-dimensional features extracted from the last layer of the EEGNet model in the PSFM dataset (A–B) Present the feature distribution domains for two-class classification tasks, comparing single-channel versus five-channel data configurations. (C–D) Display the feature distribution domains for three-class classification tasks, again contrasting single-channel against five-channel data setups.

To investigate the distribution characteristics of generated EEG signals within the feature space of classification models, we have conducted a t-SNE visualization analysis on the Bi2015a and PSFM datasets with “5.” As illustrated in Figure 12, the high-dimensional features extracted from the final layer of the EEGNet model reveal high intra-class clustering and significant inter-class separability for both binary and three-class classification tasks. The distinct class boundaries highlight the model’s exceptional discriminative capabilities. In the PSFM dataset, as depicted in Figure 13, while class boundaries generally remained clear, localized feature overlap was more pronounced compared to the Bi2015a dataset. This phenomenon suggests the similarity of certain feature dimensions across different classes in the PSFM dataset, attributable to subtle variations in brain activity during decision-making processes induced by different stimuli, resulting in relatively smaller inter-class differences. These findings further demonstrate the effectiveness of generated signals in multi-class, multi-channel classification tasks.

## Discussion

Here, we introduce a hierarchical MRM that endows generative models with human-like supervisory capabilities, fundamentally overcoming the limitations of current EEG generation techniques. Existing models, while proficient in signal synthesis, neglect the intricate temporal dynamics and functional resolution inherent in EEG signals. This oversight leads to incomplete feature representation, increasing the risks of feature redundancy, gradient explosion, and overfitting, which in turn diminishes generalization capabilities.^26^^,^^27^ To address this, we have developed a module inspired by human metacognitive processes—monitoring, regulation, and transferability—to directly integrate these functions into generative models. Our comprehensive evaluation, which goes beyond the limited metrics of previous studies, demonstrates that this module significantly enhances the diversity and similarity of synthetic EEG signals while mitigating overfitting.

The efficacy of our metacognitive module was rigorously validated using a multi-faceted framework. We quantified synthetic signal quality using MSD and SWD, and qualitatively assessed the preservation of key neurophysiological markers, such as the P300 event-related potential (ERP) and PSD. Crucially, we evaluated the practical utility of the generated data by training classifiers on mixtures of real and synthetic signals for multiclass tasks, using Acc and F1-score to probe generalization. This robust methodology confirms that our module significantly enhances model performance, particularly in architectures such as WaveGAN, effectively equipping them with the adaptability and Pre required to capture the nuanced characteristics of EEG signals across both temporal and frequency domains.

A key strength of our study lies in its validation across two distinct EEG datasets—Bi2015 and PSFM—which capture fundamentally different cognitive processes. The Bi2015 dataset, involving actively elicited P300 responses, reflects conscious cognition, whereas the PSFM dataset, capturing passively generated P300s, probes unconscious processes. This deliberate choice allowed us to test the module’s ability to model a wide spectrum of cognitive engagement. Our results show that the metacognition-enhanced models can precisely encapsulate subtle features and fluctuations, demonstrating a remarkable capacity to discern and replicate the nuances of human cognitive states. This represents a significant advance over prior work, such as that of Vahid et al.,^13^ which attempted to approximate cognitive control using a conditional GAN (cGAN) but lacked the integration of a dedicated self-monitoring and self-regulation mechanism. Consequently, their approach, tested on a single dataset with limited metrics, may not achieve the same level of similarity and robustness demonstrated by our comprehensive framework. Furthermore, their focus was on validating cognitive functions through cGAN, whereas we incorporated the metacognitive monitoring concept directly into the design of our regularizers. More importantly, in contrast to existing approaches^1^^,^^2^ that extract features from known subjects to alleviate the influence of individual differences, our method leverages EEG signals from multiple subjects to capture diverse neural responses under the same conditions. This enables the model to generalize better to unseen subjects without requiring subject-specific adaptation.

The implications of this work extend beyond synthetic data generation, offering a novel perspective on the simulation of human cognition and the future of BCIs. Current models are predominantly designed for specific tasks,^31^^,^^32^^,^^33^ inherently limiting their capacity to replicate the holistic, self-regulatory nature of human cognitive functions such as consciousness and emotion.^4^^,^^34^ Our module marks a pivotal step toward imbuing artificial systems with such generalized cognitive control. Furthermore, we propose a paradigm shift in BCI research. Instead of the conventional unidirectional mapping from EEG signals to behavior, our approach enables the reverse process: reconstructing EEG signals from cognitive intent. This generative loop provides a powerful mechanism for validating the similarity of decoded features and for probing the very nature of neural representations of intelligence.

Future research should focus on refining this cognitive alignment and streamlining its practical application. First, we will explore training paradigms that more closely mimic human learning, such as progressive reduction in training data (e.g., fewer participants or single-trial learning). This will enhance the model’s adaptability and is critical for real-time BCI deployment. Second, to address the bottleneck of manual data curation, we will develop automated algorithms for screening high-quality synthetic EEG signals. Automating this quality control is essential for scaling the approach and improving the generalization and robustness of models for large-scale cognitive computing. By pursuing these directions, we aim to fully harness the potential of metacognitive generative models to advance both fundamental neuroscience and the engineering of more intelligent, human-like neurotechnologies.

In conclusion, while cognitive activity is a vital aspect of human life, current models have yet to fully capture its complexities. Inspired by the human learning system, this study introduces a MRM for GAN-based generative models. This module, comprising three regularizers, aims to imbue these models with adaptive decision-making capabilities akin to humans. Our selection of regularizers is grounded in the metacognitive monitoring mechanism: The TR corresponds to local knowledge monitoring, the FR to global knowledge monitoring, and the latent space regularizer to the monitoring of knowledge organization strategies.

This module was rigorously tested using two meticulously chosen datasets. We first conducted an ablation study to validate the efficacy of each regularizer component. Subsequently, we integrated the module into three generative models to mitigate overfitting, thereby enhancing the diversity and similarity of the generated signals. Finally, to test the utility of our model, we conducted multiclass classification experiments using two carefully selected models. By blending generated EEG signals with real ones in varying proportions, we demonstrated that classification performance metrics reached optimal levels when the ratio of real to generated data was 1:5, indicating the effectiveness of generated data in enhancing the performance of classification models.

Based on these experimental validations, the proposed module empowers generative models to excel in neural signal simulation tasks with diverse control demands. The performance of these models aligns closely with human-like behavior in decision-making scenarios, suggesting that our module effectively mimics the human cognitive decision-making process. This advancement not only enhances the model’s ability to learn in a more human-like manner but also paves the way for more sophisticated cognitive simulations in artificial intelligence.

### Limitations of the study

The proposed module significantly enhances the capabilities of GAN-based generative models in neural signal simulation, yielding promising results. However, some limitations remain and require further investigation.

(1) Restricted dataset scope. The validation of the module in this study was confined to two specific datasets. Consequently, the generalizability of the module to other EEG datasets, such as those involving diverse cognitive tasks (e.g., SEED-VII, which comprises EEG and eye movement signals induced by 80 video clips associated with seven emotions – happiness, sadness, fear, disgust, surprise, anger, neutral and continuous emotional intensity labels), warrants comprehensive evaluation.
(2) Static regularizer weight configuration. The weights assigned to the three regularizers were predetermined based on the SWD and MS metrics, rather than being dynamically optimized during the training process. Exploring adaptive weight adjustment mechanisms, similar to those employed in the regularization algorithm based on reinforcement learning.^35^ This algorithm quantifies the importance of different actions through causal analysis and dynamically adjusts the regularization weights accordingly, prioritizing regularizers with a greater impact on model performance. Similarly, we can apply this mechanism to adjust regularization weights by quantifying the causal contribution of each regularizer to the generator’s loss function, thereby dynamically evaluating its importance. Alternatively, inspiration can be drawn from the training dynamics of token loss in language models.^36^ This method calculates the excess loss of each token between a reference model and the target model to assess the contribution of that token to model learning. Tokens with higher excess loss have a greater impact on model learning, and thus, the loss assigns higher training weights to these critical tokens, enabling the model to focus on the most valuable aspects of learning. Inspired by this, we can design an analogous “excess loss” metric for each regularizer to evaluate its contribution to the model’s learning and adjust the weights accordingly. Furthermore, an “autoguidance” method^37^ can be introduced, which uses a simplified version of the model itself to guide the generation process of diffusion models, significantly improving image quality without sacrificing diversity. Similarly, we can design a lightweight auxiliary model to calculate the loss difference between the main model and the auxiliary model in real-time, mapping this difference to the weights of the various regularization terms, thereby achieving more refined regularization control.
(3) Extrinsic integration of the metacognitive mechanism. While the proposed module exhibited a high degree of consistency with human cognitive decision-making processes in neural signal simulation, interpreted as evidence of effectively mimicking human metacognitive mechanisms, it is important to acknowledge that the module was appended as an external component to the pre-existing generator structure. Future research should investigate a more intrinsic integration of the metacognitive regulation mechanism into the core architecture of generative models, thereby providing a more robust structural foundation for claims of emulating human metacognition.

## Resource availability

### Lead contact

Requests for further information and resources should be directed to and will be fulfilled by the lead contact, Likun Xia (xlk@cnu.edu.cn).

### Materials availability

This study did not generate new unique reagents.

### Data and code availability

• Data: The Bi2015a dataset be reached at here.^35^ The perception of structure-from-motion (PSFM) are available at Science Data Bank: https://doi.org/10.57760/sciencedb.32593.
• Code: Code is publicly available at https://github.com/Seanhanyy/Metacognitive-Regulation-Module.
• Additional information: Any additional information required to reanalyze the data reported in this paper is available from the lead contact upon request.

## Acknowledgments

This work is supported by the 10.13039/501100004826Beijing Natural Science Foundation under Grant 4242033.

## Author contributions

M.Y., T.G., S.H., and L.X. conceived the project. With contributions from J.D., M.Y., T.G., S.H., and L.X. further integrated the generative model with metacognitive theory. M.Y. performed the theoretical analysis, assisted by S.H., W.Y., and C.H., M.Y., T.G., W.Y., H.C., and J.H. conducted all experiments, with C.H., S.H., and N.X. assisting in experimental design and data analysis. M.Y., T.G., L.X., and J.D. wrote the paper. During the major revision, S.H. addressed all reviewers’ comments, reorganized the manuscript structure, and drafted the supplemental information section. All authors reviewed and approved the final manuscript.

## Declaration of interests

The authors declare no competing interests.

## Declaration of generative AI and AI-assisted technologies in the writing process

During the preparation of this work, the authors used Doubao and GLM-5 to improve the English grammar and readability of the manuscript. After using this tool, the authors reviewed and edited the content as needed and take full responsibility for the content of the publication.

## STAR★Methods

### Key resources table

**Table:** 

REAGENT or RESOURCE	SOURCE	IDENTIFIER
**Deposited data**		

Bi2015a	Korczowski, L. et al.^35^	https://doi.org/10.5281/zenodo.3266930
Perception of structure-from-motion (PSFM)	Lab of Learning and Cognition, School of Psychology, Capital Normal University	Science Data Bank: https://doi.org/10.57760/sciencedb.32593

**Software and algorithms**		

Python 3.9	Python Software Foundation	https://www.python.org/

**Other**		

Code	N/A	https://github.com/Seanhanyy/Metacognitive-Regulation-Module

### Experimental model and study participant details

#### Dataset

**Bi2015a** The dataset^35^ were collected from 50 subjects (36 males and 14 females, mean age of 23.7 years). All participants self-identified their gender as consistent with their biological sex (male/female). The study was approved by the Ethical Committee of the University of Grenoble Alpes. Among 50 pairs of participants, 9 have been rejected from the study. The appearance of P300 component in this task depends on the cognitive control of the subject/brain. The data acquisition method is the recorded data under the stroboscope time of the visual task set at 50 ms, 80 ms and 110 ms, that is, the feedback/reward mechanism is used to gradually optimize the control ability of subjects and enables it to actively generate P300. In other words, the occurrence time of P300 is a random and gradual process, i.e., the time variability is large. If our generative model can actively learn such dynamic characteristics and simulate the active control of the brain, more accurate EEG signals can be generated.

**Perception of structure-from-motion (PSFM)** The dataset was collected from 22 students (10 males and 12 females, mean age of 22 years). All participants self-identified their gender as consistent with their biological sex (male/female). The Institutional Review Board (IRB) at Capital Normal University approved this study. Inclusion criteria included having normal or corrected vision (at least 20/40), right-handed, and with normal color vision. They were divided into three tasks according to different stimuli in the experiment, the stimuli for the first two tasks are called cued conditions, indicating that the direction of motion can be judged easily, i.e., one rotates to the left and another rotates to the right. The difference between them is relatively small. The third task was performed with ambiguous stimulus, where subjects could see the cue rotate either to the left or to the right. Therefore, the third tasks are different in terms of the difficulty levels of visual decision-making and more difficult than other two tasks.

#### System environment and parameter configuration

In our experiments, both Bi2015a and PSFM datasets were split into training and test sets at an 8:2 ratio. We then applied 5-fold cross-validation on the training set to further optimize model performance. Specifically, the training set was evenly divided into five subsets: in each iteration, four subsets were used for training and the remaining one for validation. By iterating through this process, the model was trained and evaluated on different combinations of subsets, which improved its stability and generalization capability.

The model was implemented in a Python environment using Keras2.3.1 with a Tensorflow2.0.0, using the TeslaP100 GPU. Batch normalization and dropout strategies were adopted to optimize the network. The initial learning rate was set to 4e−4 and weight attenuation was set to 10. The model was trained for 400 epochs using stochastic gradient descent (SGD) with exponential decay of the learning rate after each epoch and momentum. Batch size is set to 128 when the network is trained.

#### Generative adversarial module

**WaveGAN** focuses on synthesizing raw audio signals from different domains such as speech and musical instruments. It consists of similar generator and discriminator to DCGAN, but differently, WaveGAN requires one-dimensional (1D) filters instead of two-dimensional (2D) filters in DCGAN, and it optimizes the models by using the Wasserstein loss with gradient penalty (WGAN-GP). In general, WGAN-GP not only improves the training speed, but also guarantees that the gradient update is within a controllable range, so as to reduce the possibility of gradient explosion in WGAN.

**WGAN-GP** overcomes some issues in GAN,^38^ e.g., if the discriminator is too strong, the generator will appear gradient vanishing and the loss function may not be convergent; if the generator is trained well, the discriminator will appear gradient explosion. To minimize the problems, the Wasserstein distance as the error measure is used to train model, which forms a joint distribution between generated data and training data, where the lower bound of the expected data is selected to optimize the model. A gradient penalty (GP) is set instead of weight cutting to satisfy the first-order Lipschitz function constraint of the discriminator loss, thus enhancing the controllability of the gradient during optimization.

**CWGAN-GP** is a Class-Conditioned WGAN-GP that integrates a classifier into WGAN-GP.^39^ Class tags are included in the generator, which help mitigate schema crashes. It guides the data generation process by providing additional inputs to the generator and discriminator, such as category labels or specific features, so that the generated data is not only real but also meets specific conditions, thereby improving the flexibility of GAN applications and the controllability of generated data.

### Method details

#### Metacognitive regulation module (MRM)

The metacognitive regulation module incorporates three specialized regularizers: frequency domain regularizer, time domain regularizer and latent space domain regularizer. Each of these regularizers is designed to refine the model’s generation mechanism, as outlined in Equation 1, where λ represents a hyperparameter that tunes the trade-off between similarity and diversity. and its specific value for λ is determined through a detailed process, which is elaborated upon in “selection of hyperparameter” section. In the context of the adversarial network, we retain the conventional loss function, as depicted in Equation 2, to ensure the model’s ability to engage in the adversarial training process effectively.

(Equation 1) $$\begin{array}{c} L_{G_{final}}=L_{G}+\lambda \frac{1}{L_{SR}}+\left(1-\lambda \right)\left(\frac{1}{2}L_{FR}+\frac{1}{2}L_{TR}\right) \end{array}$$

(Equation 2) $$\begin{array}{c} \min_{G}\max_{D}V\left(D,G\right)=E_{x∼p_{data}}\left[\log\;D\left(x\right)\right]+E_{z∼p_{z}\left(z\right)}\left[\log\left(1-D\left(G\left(z\right)\right)\right)\right] \end{array}$$

**Time domain Regularizer.** It narrows the gap between real and generated EEG signals by calculating the curvatures in time domain, so as to improve the similarity. Curvature refers to the amount by which a curve deviates from being a straight line or a flat surface. It quantifies the degree of bending or curving at a specific point on a curve or surface. In mathematical terms, curvature is defined as the reciprocal of the radius of curvature at a given point. The greater the curvature is, the more curved the signal is, i.e., the higher frequency. EEG signal is a set of discrete-time sampling points, for example, (*x*_1_, *y*_1_), (*x*_2_, *y*_2_), (*x*_3_, *y*_3_), It indicates that the middle point (*x*_2_, *y*_2_) is used as the curvature estimate of the curve. The parametric equation is expressed as seen in Equation 3, where both a and b are parameters of t.

(Equation 3) $$\begin{array}{c} \left\{ \begin{array}{c} x=a_{1}+a_{2}t+a_{3}t^{2}\\ y=b_{1}+b_{2}t+b_{3}t^{2} \end{array}\right. \end{array}$$

After that, the curvature is obtained in Equation 4, where k presents curvature, *x*′ and *x*″, *y*′ and *y*″ accordingly indicate first and second derivatives of x and y.

(Equation 4) $$\begin{array}{c} k=\frac{\left|x^{\prime \prime }y^{\prime }-x^{\prime }y^{\prime \prime }\right|}{{\left({\left(x^{\prime }\right)}^{2}+{\left(y^{\prime }\right)}^{2}\right)}^{\frac{3}{2}}}=\frac{2\left(a_{3}b_{2}-a_{2}b_{3}\right)}{{\left(a_{2}^{2}+b_{2}^{2}\right)}^{\frac{3}{2}}} \end{array}$$

We then calculate the curvature of the real EEG and generated EEG signals in time domain, where the curvature calculates *L*_*TR*_, as shown in Equation 5, where *k*^*real*^ and *k*^*fake*^ represent the curvature of real and generated EEG signals, respectively.

(Equation 5) $$\begin{array}{c} L_{TR}=-E_{k^{real}∼p_{data},k^{fake}∼p_{G}}\lfloor k^{real}\cdot \log\left(k^{fake}\right)+\left(1-k^{real}\right)\cdot \log\left(1-k^{fake}\right)\rfloor \end{array}$$

TR adopts the binary cross entropy to minimize the gap between the real EEG and generated EEG signals, so as to improve the similarity of generated EEG signals in time domain.

**Frequency domain Regularizer.** It calculates the ratio of PSD between the real and generated EEG signals. In order to obtain PSD of the signals, we initially calculate its DFT, which discretizes the signals into the discrete frequency domain, as shown in Equation 6:

(Equation 6) $$\begin{array}{c} F\left[k\right]=\sum_{n=0}^{N-1}x\left[n\right]e^{-j\frac{2\pi }{N}kn} \end{array}$$

where *x*[*n*] is discrete input signal, *n* = 0,1, …,*N*-1, and *N* presents the number of sampling points; *F*[*k*] denotes output result of DFT, *k* = 0,1, …,*N*-1, indicates imaginary axis.

We then apply the DFT to the loss function *L*_*FR*_, as seen in Equation 7. It calculates the binary cross entropy between the output *F*^*fake*^ of the generated EEG and the average *F*^*real*^ obtained from the real EEG signal. FR minimizes the discrepancies between two EEG signals by measuring the probability distribution of the signal in the frequency domain, that is, to improve the similarity of the generated EEG.

(Equation 7) $$\begin{array}{c} L_{FR}=-E_{F^{real}∼p_{data},F^{fake}∼p_{G}}\lfloor F^{real}\cdot \log\left(F^{fake}\right)+\left(1-F^{real}\right)\cdot \log\left(1-F^{fake}\right)\rfloor \end{array}$$

**Latent space Regularizer.** It improves the diversity of generated signals by calculating the proportion of input noise and generated EEG signals, as shown in Equation 8, where *z*_1_ and *z*_2_ are random noise, and *G*(*z*_1_) and *G*(*z*_2_) present corresponding generated EEG signals.^31^

(Equation 8) $$\begin{array}{c} L_{SR}=E_{x∼p_{g}}\left[\frac{1-\cos\left(G\left(z_{1}\right),G\left(z_{2}\right)\right)}{1-\cos\left(z_{1},z_{2}\right)}\right] \end{array}$$

The numerator represents the similarity between *G*(*z*_1_) and *G*(*z*_2_), while the denominator indicates the similarity between *z*_1_ and *z*_2_. Some studies have shown that the more similar *z*_1_ and *z*_2_ are, the higher chances *G*(*z*_1_) and *G*(*z*_2_) fall into the same mode.^10^^,^^40^ When *z*_1_ and *z*_2_ are very similar, indicating a small value in the denominator, i.e., the above equation signifies dissimilarity between *G*(*z*_1_) and *G*(*z*_2_), thus enhancing the diversity of the generated EEG signals.

#### Theoretical analysis of generalization ability and generator gradient

The goal of GAN is to train a generator capable of deceiving a discriminator by minimizing the distance between the empirical distribution and the generated distribution. Typically, we assume an infinite true distribution *P*^∗^ and a generated distribution *P*^*g*^. However, in real-world scenarios, we are usually limited to processing a dataset of size n. We refer to the theoretical part of Mbacke et al.,^32^ considering a metric space (X, d), where there is an unknown distribution *P*^∗^, and the training set *S* = *x*_1_, …,*x*_*n*_ is independently and identically distributed from *P*^∗^. We denote $P_{n}^{*}$ has the empirical distribution constructed from the training set S, used as an approximation of *P*^∗^. At the same time, a hypothetical space G is introduced, where each generator *g*∈*G* induces a distribution *P*^*g*^ on the space X. From this distribution *P*^*g*^, a generated sample set $S_{g}=\left\{{\hat{x}}_{1},\ldots ,{\hat{x}}_{n}\right\}∼P^{g⊗n}$ can be obtained.

For a given generator *g*∈*G*, we define the corresponding empirical risk as shown in Equation 9.

(Equation 9) $$\begin{array}{c} W_{F}\left(P_{n}^{*},P_{n}^{g}\right)=E_{S_{g}}\left[d_{F}\left(P_{n}^{*},P_{n}^{g}\right)\right] \end{array}$$

Here, the expectation is calculated with respect to the sample set S from the generator distribution $P_{n}^{*}$. At the same time, $d_{F}\left(P_{n}^{*},P_{n}^{g}\right)$ represents the cumulative probability measure induced by the discriminator*f*∈*F*. The generalization error is defined as seen in Equation 10, i.e., the difference between the overall risk and the empirical risk.

(Equation 10) $$\begin{array}{c} {ℇ}_{gan}=E_{S∼P^{g⊗n}}\left[W_{F}\left(P_{n}^{*},P_{n}^{g}\right)\right]-W_{F}\left(P_{n}^{*},P_{n}^{g}\right) \end{array}$$

Based on the above definition, to evaluate the impact of the frequency domain regularizer and the time domain regularizer on the model’s generalization error, we use VC dimension (Vapnik-Chervonenkis Dimension) theory to analyze the upper bound of the generalization error of the GAN.

Let *F*⊆*Lip*_1_ be a symmetric set of real-valued functions defined on the space *X*, *P*^∗^ be the true signal distribution, and *S*∈*X* represent a sample set obtained from *P*^∗^ with independent and identically distributed samples. Suppose the generator *g*∈*G* generates a probability distribution *P*^*g*^ on X, *d*_*VC*_(*G*) represents the VC dimension of the hypothesis space G, and N represents the sample size. Under the above assumptions, for a given confidence level parameter *δϵ*(0,1), there exists a constant *C* > 0 such that the following inequality holds with a probability of at least 1-*δ*.

(Equation 11) $$\begin{array}{c} E_{S}\left[W_{F}\left(P_{n}^{*},P^{g}\right)\right]-W_{F}\left(P_{n}^{*},P^{g}\right)\leq C\left(\sqrt{\frac{d_{VC}\left(G\right)\log\left(N\right)+\log\left(\frac{1}{\delta }\right)}{N}}\right) \end{array}$$

After introducing the frequency domain regularizer (FR) and the time domain regularizer (TR) to the original hypothesis space *G*, and by constraining EEG signals in the frequency and time domains, the effective hypothesis space is reduced to *G*_*FR*,*TR*_ as shown in Equation 12:

(Equation 12) $$\begin{array}{c} G_{FR,TR}=\left\{g\in G|L_{FR}\left(g\right)\leq {ℇ}_{1},L_{TR}\left(g\right)\leq {ℇ}_{2}\right\} \end{array}$$

where *ℇ*_1_ and *ℇ*_2_ represent the constraint thresholds for the frequency domain regularizer and the time domain regularizer, respectively. Let *N*(*G*,Δ) denote the minimum number of balls of radius Δ required to cover the hypothesis space, i.e., the covering number of the hypothesis space. Then, the constrained hypothesis space *G*_*FR*,*TR*_ satisfies: *logN*(*G*_*FR*,*TR*_). Since the VC dimension is positively correlated with the covering number of the hypothesis space^79^, after adding the frequency domain regularizer (FR) and the time domain regularizer (TR), *d*_*VC*_(*G*_*FR*,*TR*_)≤*d*_*VC*_(*G*). Therefore, it can be concluded as seen in Equation 13.

(Equation 13) $$E_{S}\left[W_{F}\left(P_{n}^{*},P^{g}\right)\right]-W_{F}\left(P_{n}^{*},P^{g}\right)\leq C\left(\sqrt{\frac{d_{VC}\left(G_{FR,TR}\right)\log\left(n\right)+\log\left(\frac{1}{\delta }\right)}{n}}\right)\leq C\left(\sqrt{\frac{d_{VC}\left(G\right)\log\left(n\right)+\log\left(\frac{1}{\delta }\right)}{n}}\right)$$

This means that when the FR and the TR are introduced, the upper bound on the generalization error is further tightened.

Based on the theoretical derivation,^33^ it is found that the average gradient norm of the generator *G* corresponding to the latent space regularizer on the interval [*z*_1_,*z*_2_] has the lower bound as shown in Equation 14.

(Equation 14) $$\begin{array}{c} E_{z_{1},z_{2}}\left[\frac{1-\cos\left(G\left(z_{1}\right),G\left(z_{2}\right)\right)}{1-\cos\left(z_{1},z_{2}\right)}\right]\leq E_{z_{1},z_{2}}{\left[\int_{0}^{1}\|\nabla_{z}G\left(\gamma \left(t\right)\right)\|dt\right]}^{2} \end{array}$$

Here, *γ*(*t*) = *tz*_1_+(1-*tz*_2_), and this formula constructs the connection between *z*_1_ and *z*_2_ linearly. This implies that during the optimization of the latent space regularizer, the gradient norm of the generator ‖∇_*z*_*G*‖ will be increased, thereby promoting the generator *G*(*x*,*z*) to achieve broader sample coverage in the output space under the condition of fixed constraints. In this way, the generator can obtain more informative gradient signals from the discriminator, enhancing the quality and diversity of generation. The specific proof process is shown in Derivation of the lower bound of the generator’s gradient norm.

#### Multifaceted assessment

Our study investigates the proposed module across various tasks. Firstly, an ablation study was conducted to validate the effectiveness of the designed regularizers using MS and SWD. Secondly, three representative generative models were evaluated to assess their similarity and diversity through visual analysis using P300 and PSD. Thirdly, the module was assessed by using the generated signals from step (2) to optimize two carefully selected classifiers. The results were analyzed both quantitatively and qualitatively, using different metrics and t-SNE visualization for the classification tasks.

#### Qualitative analysis

To assess the similarity and diversity of the generated EEG signals, we conducted a comprehensive analysis using both temporal and spectral characteristics. Specifically, we examined the P300 and PSD. The P300 ERP^36^^,^^37^ is characterized by a positive deflection in the EEG signal occurring approximately 300–500 ms post-stimulus. Temporal similarity and diversity were evaluated by determining the presence and characteristics of the P300 in the generated signals. Furthermore, we employed Welch’s method for PSD estimation to analyze the energy distribution of the generated EEG signals across different frequency bands, thereby assessing their quality in the frequency domain.

#### Quantitative analysis

**Generation indicators** Mode Score (MS)^41^ and Sliced Wasserstein distance (SWD) are applied to analyze generated signals. MS evaluates diversity by calculating the cross entropy between real and generated signals label distribution, and higher MS indicates better diversity. SWD^42^ gauges the similarity by calculating Wasserstein-1 distance between real and generated signals, and lower SWD suggests greater similarity.

**Classification metrics** The following metrics were calculated to facilitate better observation and objective evaluation of the classification method: accuracy $Acc=\frac{TP+TN}{TP+FP+TN+FN}$, precision $Pre=\frac{TP}{TP+FP}$, recall $Rec=\frac{TP}{TP+FN}$, F1 Score $F1=\frac{2TP}{2TP+FN+FP}$, and area under curve $AUC=\frac{\sum I\left(P_{positive},P_{negative}\right)}{MXN}$, where *M* is the number of positive samples and *N* denotes the number of negative samples.

#### Derivation of the lower bound of the generator’s gradient norm

In this section, we present a detailed derivation of the lower bound for the generator’s gradient norm.

(1) When applying latent spatial regularization, we normalize both the generated vector and the noise vector, e.g., norms = 1. The normalization allows the cosine distance to be expressed equivalently as the Euclidean distance, which yields the results in both Equation 15 and Equation 16.

(Equation 15) $$\begin{array}{c} 1-\cos\left(z_{1},z_{2}\right)=\frac{{\left\|z_{1}-z_{2}\right\|}^{2}}{2} \end{array}$$

(Equation 16) $$\begin{array}{c} 1-\cos\left(G\left(z_{1}\right),G\left(z_{2}\right)\right)=\frac{{\left\|G\left(z_{1}\right)-G\left(z_{2}\right)\right\|}^{2}}{2} \end{array}$$

Where *z*_1_ and *z*_2_ represent noise vectors, and *G*(*z*_1_) and *G*(*z*_2_) represent the sample vectors generated by the generator with *z*_1_ and *z*_2_ as inputs, respectively.

(2) We start from this regularizer and consider its impact on the gradient norm of the generator. For any latent variables *z*_1_ and *z*_2_ sampled from a Gaussian distribution, we apply the Gradient Theorem to the linear interpolation path.^33^ This yields a bound on the gradient norm Equation 17.

(Equation 17) $$\frac{1-\cos\left(G\left(z_{1}\right),G\left(z_{2}\right)\right)}{1-\cos\left(z_{1},z_{2}\right)}={\left[\frac{\left\|G\left(z_{1}\right)-G\left(z_{2}\right)\right\|}{\left\|z_{1}-z_{2}\right\|}\right]}^{2}={\left[\frac{\left\|\int_{\gamma \left[z_{1},z_{2}\right]}\nabla_{z}G\left(z\right)dz\right\|}{\left\|z_{1}-z_{2}\right\|}\right]}^{2}={\left[\frac{\left\|\int_{0}^{1}\nabla_{z}G\left(\gamma \left(t\right)\right)\gamma^{\prime }\left(t\right)dt\right\|}{\left\|z_{1}-z_{2}\right\|}\right]}^{2}={\left[\frac{\left\|\int_{0}^{1}\nabla_{z}G\left(\gamma \left(t\right)\right)\cdot \left(z_{1}-z_{2}\right)dt\right\|}{\left\|z_{1}-z_{2}\right\|}\right]}^{2}\leq {\left[\frac{\left\|\int_{0}^{1}\left\|\nabla_{z}G\left(\gamma \left(t\right)\right)\right\|\left\|\left(z_{1}-z_{2}\right)\right\|dt\right\|}{\left\|z_{1}-z_{2}\right\|}\right]}^{2}\leq {\left[\int_{0}^{1}\left\|\nabla_{z}G\left(\gamma \left(t\right)\right)\right\|dt\right]}^{2}$$

We then take the expectation from both sides with respect to *z*_1_ and *z*_2_, thus obtaining Equation 18.

(Equation 18) $$\begin{array}{c} E_{z_{1},z_{2}}\left[\frac{1-\cos\left(G\left(z_{1}\right),G\left(z_{2}\right)\right)}{1-\cos\left(z_{1},z_{2}\right)}\right]\leq E_{z_{1},z_{2}}{\left[\int_{0}^{1}\|\nabla_{z}G\left(\gamma \left(t\right)\right)\|dt\right]}^{2} \end{array}$$

#### Supplementary on experimental results and analysis

##### Selection of hyperparameter

To select the optimal *λ*, we evaluate two normalized metrics: MS and SWD. Both are standardized via *Z* score normalization to align their scales.

For each candidate *λ*, the relative variations of MS and SWD relative to the original model are computed by Equation 19–Equation 20.

(Equation 19) $$\begin{array}{c} \Delta MS={MS}_{\lambda }-{MS}_{original},\lambda \in \left(0,1\right) \end{array}$$

(Equation 20) $$\begin{array}{c} \Delta SWD={SWD}_{original}-{SWD}_{\lambda },\lambda \in \left(0,1\right) \end{array}$$

Note that a positive Δ*MS* indicates improved diversity, while a positive Δ*SWD* actually implies worse similarity (since lower SWD is better). However, in our empirical analysis, we observed that for certain *λ*, both differences can be interpreted as improvements when considered jointly after normalization—specifically, when both normalized differences are positive, reflecting a favorable trade-off.

We therefore consider values of *λ* for which both Δ*MS*>0 and Δ*SWD*>0. The optimal *λ* is the one that maximizes the total gain, which is defined as shown in Equations 21 and 22:

(Equation 21) $$\begin{array}{c} \Delta Sum=\Delta MS+\Delta SWD\;s.t.\;\Delta MS>0\cap \Delta SWD>0 \end{array}$$

(Equation 22) $$\begin{array}{c} \lambda =\mathrm{argmax}\left(\Delta Sum\right) \end{array}$$

Taking the WaveGAN model as an example the public dataset, where the data were divided into training and test sets in a ratio of 8:2, and the training data were further divided into training and validation sets using the 5-fold cross-validation (CV) method, the results are shown in Figure S1. It is observed that both Δ*MS* and Δ*SWD* are positive values when *λ* is set to 0.6. Therefore, the maximum Δ*Sum* and the optimal value for *λ* are achieved, i.e., *λ* = 0.6.

### Quantification and statistical analysis

#### Visual analysis through juxtaposition of temporal and spectral maps

In this section, we investigate the EEG signals generated by WGAN-GP (MetacWGAN) and CWGAN-GP (MetacCWGAN) models with embedded metacognitive regulation module. We conduct a visual comparison analysis of the temporal and spectral maps against real EEG signals. Figures S2 and S3 present the time domain visualization results from the Bi2015a and PSFM datasets. Overall, the EEG signals generated by the models incorporating the metacognitive regulation module exhibited a more pronounced similarity to real EEG signals, particularly in terms of the P300.

In the Bi2015a dataset, as indicated by the red box, the MetacWGAN (Figure S2B) exhibits a distinct positive waveform peak within the 300–500 ms time window. In contrast, the red trend line reveals that the positive peak of the WGAN-GP (Figure S2C) shows a relatively flatter variation during the same time interval. Similarly, within the region marked by the red box, the MetacCWGAN (Figure S2D) demonstrates a significantly greater signal fluctuation amplitude around 300 ms compared to the CWGAN-GP (Figure S2E). These observations suggest that the metacognitive regulation module enhances the GAN’s ability to capture the key characteristics of the P300 in real EEG signals. In the PSFM dataset, although the generated EEG signals still show some discrepancies in smoothness compared to the real signals, the red trend lines clearly highlight the P300. As indicated by the red box, the MetacWGAN (Figure S3B) exhibits a distinct negative deflection around 300 ms. In contrast, the red trend line shows that the WGAN-GP (Figure S3C) displays a relatively flat waveform during the same time window. Similarly, within the 300–500 ms time window (as marked by the red box), the MetacCWGAN (Figure S3D) demonstrates a significant positive peak, whereas the CWGAN-GP (Figure S3E) shows a relatively flat waveform during the same period. These observations indicate that the metacognitive regulation module enables the GAN to more effectively capture the key characteristics of the P300 in real EEG signals.

Figures S4 and S5 present the frequency domain visualization results for the Bi2015a and PSFM datasets. Overall, the EEG signals generated by models incorporating the metacognitive regulation module exhibit a higher similarity to real EEG signals in terms of PSD. In the Bi2015a dataset, MetacWGAN (Figure S4B) shows distinct spectral peak characteristics within the 20–30 Hz frequency band (as indicated by the red box), whereas the WGAN-GP (Figure S4C) exhibits little to no energy fluctuation in this band. Similarly, the MetacCWGAN (Figure S4D) also demonstrates significant energy fluctuations within the 20–30 Hz band (as marked by the red box), while the CWGAN-GP (Figure S4E) shows relatively flat energy changes in the same frequency range. These results further confirm the effectiveness of the metacognitive regulation module in enhancing the realism of the generated EEG signals in the frequency domain. In the PSFM dataset, the PSD of the real EEG signals (Figure S5A) exhibits a stable energy distribution pattern. The EEG signals generated by MetacWGAN (Figure S5B) also demonstrate a relatively consistent and stable energy trend. In contrast, the energy trend of the EEG signals generated by WGAN-GP(Figure S5C) shows a significant deviation from the gradually decreasing energy pattern observed in real brain signals. Moreover, compared to the CWGAN-GP (Figure S5E), the metacognitive MetacCWGAN (Figure S5D) exhibits an energy trend that is more consistent with the characteristics of real signals. These results further confirm the effectiveness of the metacognitive regulation module in enhancing the authenticity of the frequency-domain features of the generated signals.

#### Analysis of trends in model accuracy improvement

To verify whether the model’s accuracy reaches saturation, we conducted additional experiments with a 1:7 split ratio following the steps below.

(1) Formulation of an objective function using a power function as seen in Equation 23, where the parameters *a*, *γ* and *b* denote the learnable parameters in the function, $x={\left[x_{1},x_{2},\ldots ,x_{i}\right]}^{T}$ presents the proportion of generated EEG signals to the real ones and $y={\left[y_{1},y_{2},\ldots ,y_{i}\right]}^{T}$ indicates the average accuracy for classification.

(Equation 23) $$\begin{array}{c} y=ax^{\gamma }+b,a,b,\gamma \in R \end{array}$$

(2) Optimization of the function using the least squares method as seen in Equation 24, where *S*(*a*,*b*,*γ*) denotes the sum of squared residuals between the observed model accuracy *y*_*i*_ and the values predicted by $ax_{i}^{\gamma }+b$.

(Equation 24) $$\begin{array}{c} S\left(a,b,\gamma \right)=\sum_{i=1}^{n}{\left(y_{i}-ax_{i}^{\gamma }-b\right)}^{2} \end{array}$$

To obtain the parameters of the model, we conducted six experiments by varying the proportion of generated EEG data relative to real data. In each experimental setting, we adjusted the ratio of generated to real EEG signals by setting the number of generated signals per real signal to *x* = 0,1,2,3,4,5. Specifically, when *x* = 0, the model is trained exclusively on real signals; *x* = 5 indicates that the ratio of real EEG signals to the generated EEG signals is 1:5. The variable y is the average accuracy for classification on the dataset for each mixture.

This yields five data points: (*x*,*y*)=(1,*y*_1_),(2,*y*_2_),(3,*y*_3_),(4,*y*_4_),(5,*y*_5_), where each *y*_*i*_ is the mean accuracy obtained from repeated trials under the corresponding mixing ratio. These points were fitted to Equation 24 by minimizing the sum of squared residuals, yielding the optimal parameter estimates and resulting in the fitted power function as seen in Equation 25.

(Equation 25) $$\begin{array}{c} y=8.6x^{0.40}+79.6 \end{array}$$

Finally, we conduct the experiment at a 1:7 ratio of *x*, and the results are updated in Figure S6. It is observed that while the accuracy increased from the 1:5 baseline, the improvement margin narrowed significantly (from 95.96% to 96.48%). This trend confirms our hypothesis that while increasing generated data boosts performance, it eventually leads to a saturation point where the benefits diminish, validating the practical limit of data augmentation for this task.

#### Performance evaluation of the MRM in Diffusion-TS models

To further evaluate the generalization ability of our proposed MRM beyond GANs, we extended our experiments to a diffusion-based framework. Specifically, we selected the Diffusion-TS model due to its state-of-the-art performance in capturing temporal and spectral features in EEG signals. We incorporated the module into the training pipeline of Diffusion-TS using the Bi2015a dataset, following the configuration described in the “system environment and parameter configuration” section.

The experimental results are illustrated in Figure S7. For the WaveGAN model, the baseline MS score is 1.4357, while our approach incorporating MRM achieves an MS score of 1.8672. For the Diffusion-TS model, our approach yields a substantial improvement in MS, rising from 2.02 to 5.6477. These improvements across both models demonstrate that MRM effectively enhances the diversity of the generated signals. These results can be attributed to the SR mechanism, which imposes a penalty on the inconsistency between the dissimilarity of generated EEG signals and the dissimilarity ratio of their corresponding latent noises. This constraint drives the model to learn a more complete feature distribution.

Regarding the SWD score, for the WaveGAN model, the baseline is 0.0116, while our approach achieves a lower score of 0.0065. However, for the Diffusion-TS model, incorporating MRM results in a slight increase in SWD, rising from 0.5375 to 0.575. These findings indicate that while MRM effectively increases the similarity between generated and real signals in the WaveGAN framework, it reduces this similarity in the Diffusion-TS context. This phenomenon occurs because diffusion models tend to produce signals that cluster around the mean of local data, which easily leads to over-smoothing of signals and the loss of valuable details in EEG signals.^43^ Consequently, the baseline SWD of the Diffusion-TS model is significantly higher than that of WaveGAN. Furthermore, diffusion models generate signals through a fixed, iterative denoising process,^44^ which dynamically adjusts parameters based on the previous state. By incorporating TR and FR, the model is encouraged to maintain similarity with real signals in both the temporal and frequency domains. However, given that real EEG signals contain non-stationary transient details, imposing such strict constraints throughout the multi-step iterative process may progressively suppress or filter out these details, thereby reducing the fidelity of the generated signals.

#### Analysis of experiment classification results

To further demonstrate the effectiveness of the meta-cognitive regulation module, this section explores how generated EEG signals enhance the performance of classification models. Figures S8–S23 present results from two-class and three-class classification tasks on the Bi2015a and PSFM datasets using the EEGNet and DsMuLB-HiTA models in both single-channel and five-channel configurations. The experimental results demonstrate that as the training data size increases, the performance metrics of the models generally improve significantly and reach their optimal levels when the ratio of generated data is 5. Taking the metacWGAN as an example, when applying the EEGNet model on the Bi2015a dataset, increasing the amount of generated signals to five times the original data led to an accuracy improvement of 10.6% under the single-channel configuration, and 12.79% under the five-channel configuration. In the three-class classification task, the performance gains were even more pronounced, with accuracy increases of 15.14% for single-channel and 15.48% for five-channel configurations.

Overall, the performance gains from five-channel data were generally higher than those from single-channel data, especially in more complex classification tasks where the difference was more significant. For instance, in the PSFM dataset, the DsMuLB-HiTA model achieved a 36.87% accuracy improvement in the five-channel three-class task (Figure S8A) after incorporating generated signals at five times the original amount, demonstrating the effectiveness of generated signals in complex tasks. It is worth noting that the accuracy using only real data in this task was merely 55.23%, likely due to insufficient training data volume in complex tasks with multi-channel configurations. After adding generated data, all classification metrics improved significantly, further confirming the practical value of generated data in such scenarios.

Regarding model comparison, EEGNet showed stable performance with low error variance (standard deviation *SD* ≤ 0.6%) in relatively simple tasks like binary classification. In contrast, DsMuLB-HiTA, leveraging its bidirectional LSTM and attention mechanisms, exhibited stronger feature extraction capabilities in complex tasks such as multi-class classification with multi-channel inputs.

The introduction of generated signals not only improved the absolute values of model performance metrics but also reduced the standard deviation of model outputs, indicating enhanced model robustness. However, in some cases, the standard deviation increased; for example, in Figure S8B, when the amount of generated signals increased to five times, the model output’s standard deviation showed a certain degree of rise. This phenomenon may be due to the quality of generated samples not improving in tandem with their quantity, potentially introducing distribution shifts or redundant information that affect the model’s generalization ability.

Additionally, in most tasks, the MetacWaveGAN demonstrated the best performance, which may be attributed to its structural design tailored for time-series data, making it more suitable for modeling and generating EEG signals.
