# Supplementary material for: Integrating metacognitive mechanisms optimizes EEG generative models via hierarchical regularization
Source: iScience. 2026 Apr 16;29(6):115785. doi: 10.1016/j.isci.2026.115785 (PMC13233779; doi:10.1016/j.isci.2026.115785)
Supplement: Document S1. Figures S1–S23 [file mmc1.pdf]

iScience, Volume *29*

Supplemental information

Integrating metacognitive mechanisms optimizes EEG generative models via hierarchical regularization

Miaomiao Yu, Te Guo, Shangen Han, Na Xue, Wanying Yang, Junda Huang, Hongyu Chen, Cheng He, Jinhong Ding, and Likun Xia

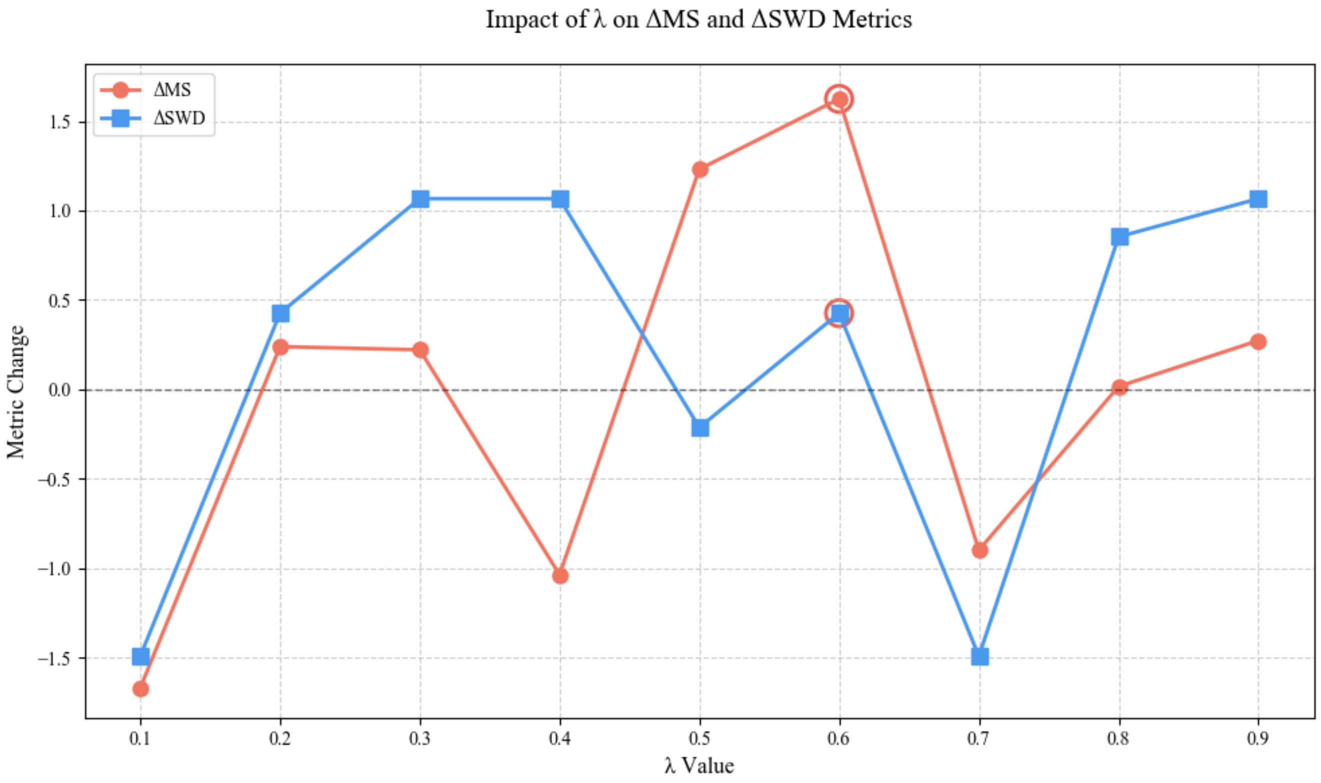

Figure S1: The impact of hyperparameter λ

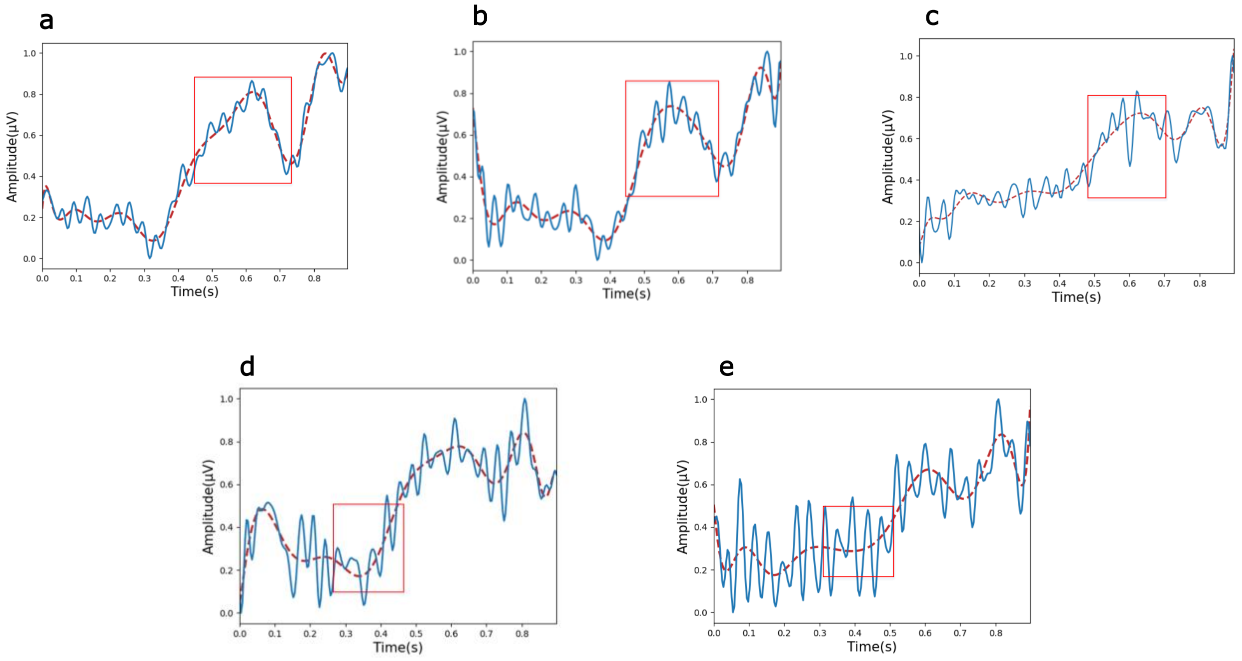

Figure S2: Comparison of real EEG and generated EEG signals from Bi2015a dataset. **(a)**.Display of Bi2015a signals. **(b)-(c)**.Signals generated by MetacWGAN and GAN, respectively. **(d)-(e)**.Signals generated by MetacCWGAN and GAN respectively. The solid line represents the EEG signal, while the dashed line corresponds to its fitted trend line

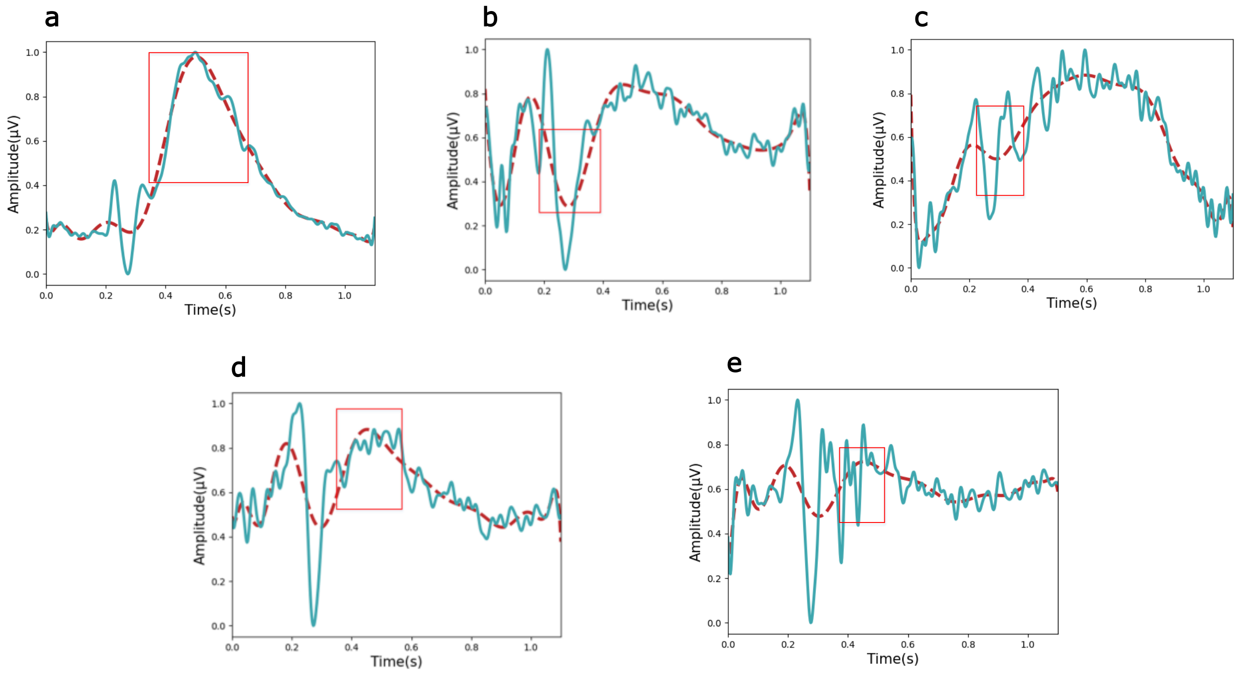

Figure S3: Comparison of real EEG and generated EEG signals from PSFM dataset. **(a)**.Display of PSFM signals. **(b)-(c)**.Signals generated by MetacWGAN and GAN, respectively. **(d)-(e)**.Signals generated by MetacCWGAN and GAN respectively. The solid line represents the EEG signal, while the dashed line corresponds to its fitted trend line

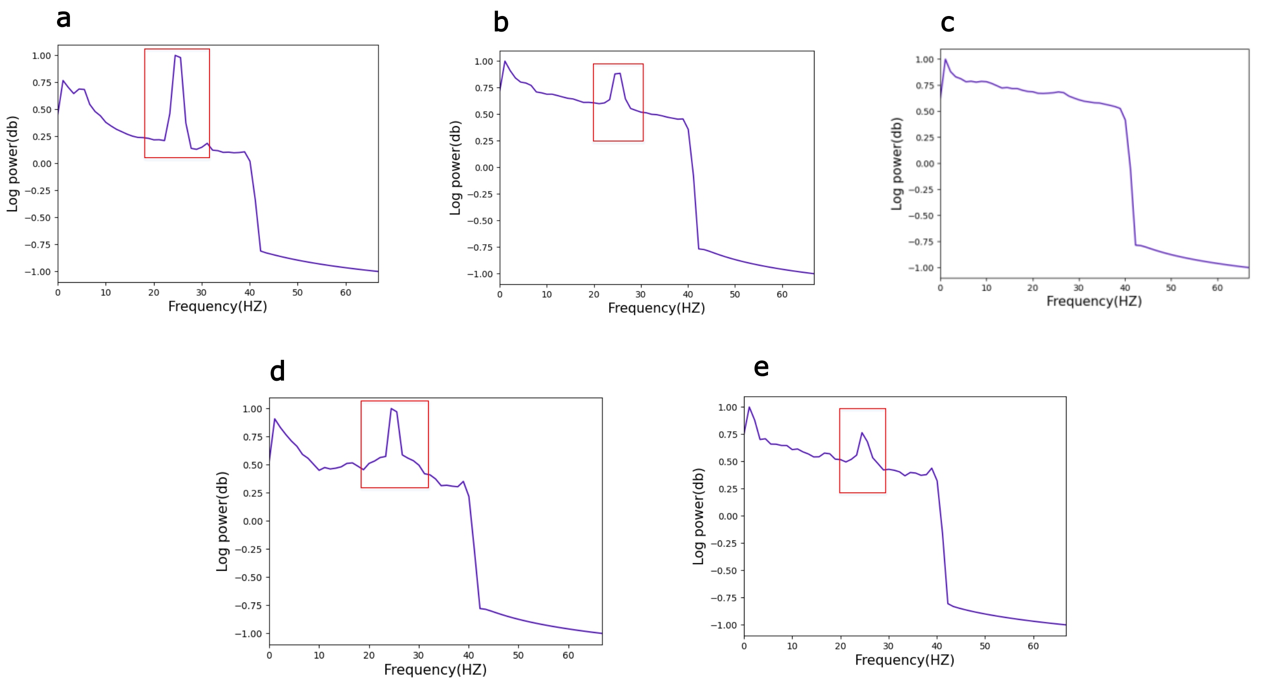

Figure S4: Comparison of PSD between real EEG and generated EEG signals from Bi2015a dataset. **(a)**.Display of Bi2015a signals. **(b)** and **(c)**.Present the signals generated by MetacW-GAN and GAN, respectively. **(d)-(e)**.Signals generated by MetacCWGAN and GAN, respectively.

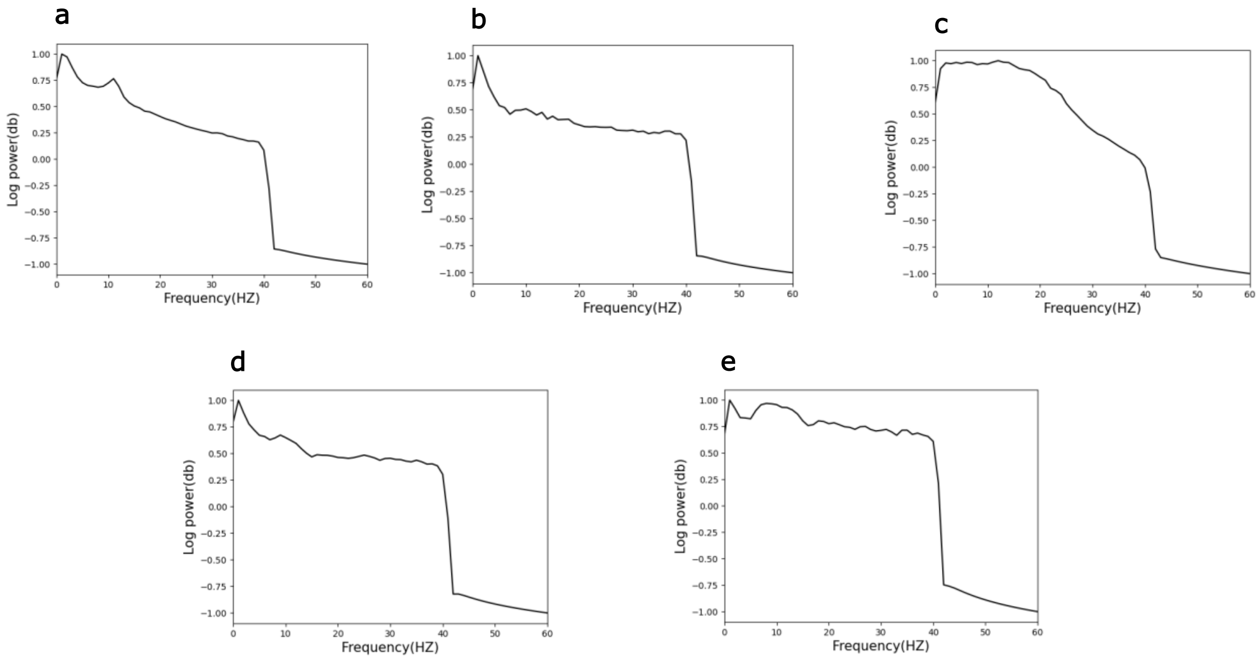

Figure S5: Comparison of PSD between real EEG and generated EEG signals from PSFM dataset. **(a)**.Display of PSFM signals. **(b)** and **(c)**.Present the signals generated by MetacWGAN and GAN, respectively. **(d)-(e)**.Signals generated by MetacCWGAN and GAN, respectively.

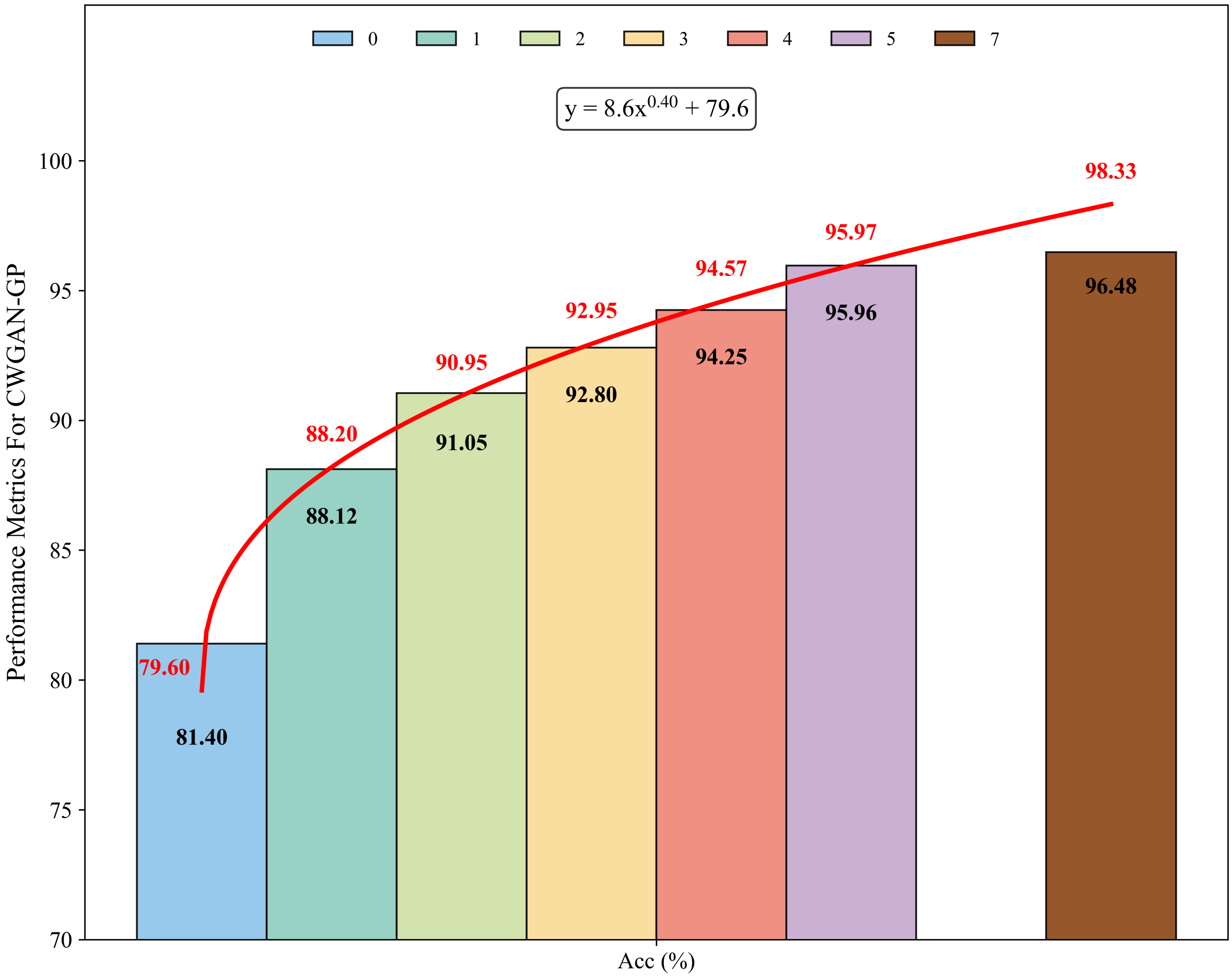

Figure S6: Fitting performance of the proposed power function with different proportion of generated data. The x-axis indicates the ratio of generated data, and the y-axis indicates model accuracy (%). Bars represent experimental accuracy associated the ratio, and the red line denotes the power function $y = 8.6x^{0.4} + 79.6$.

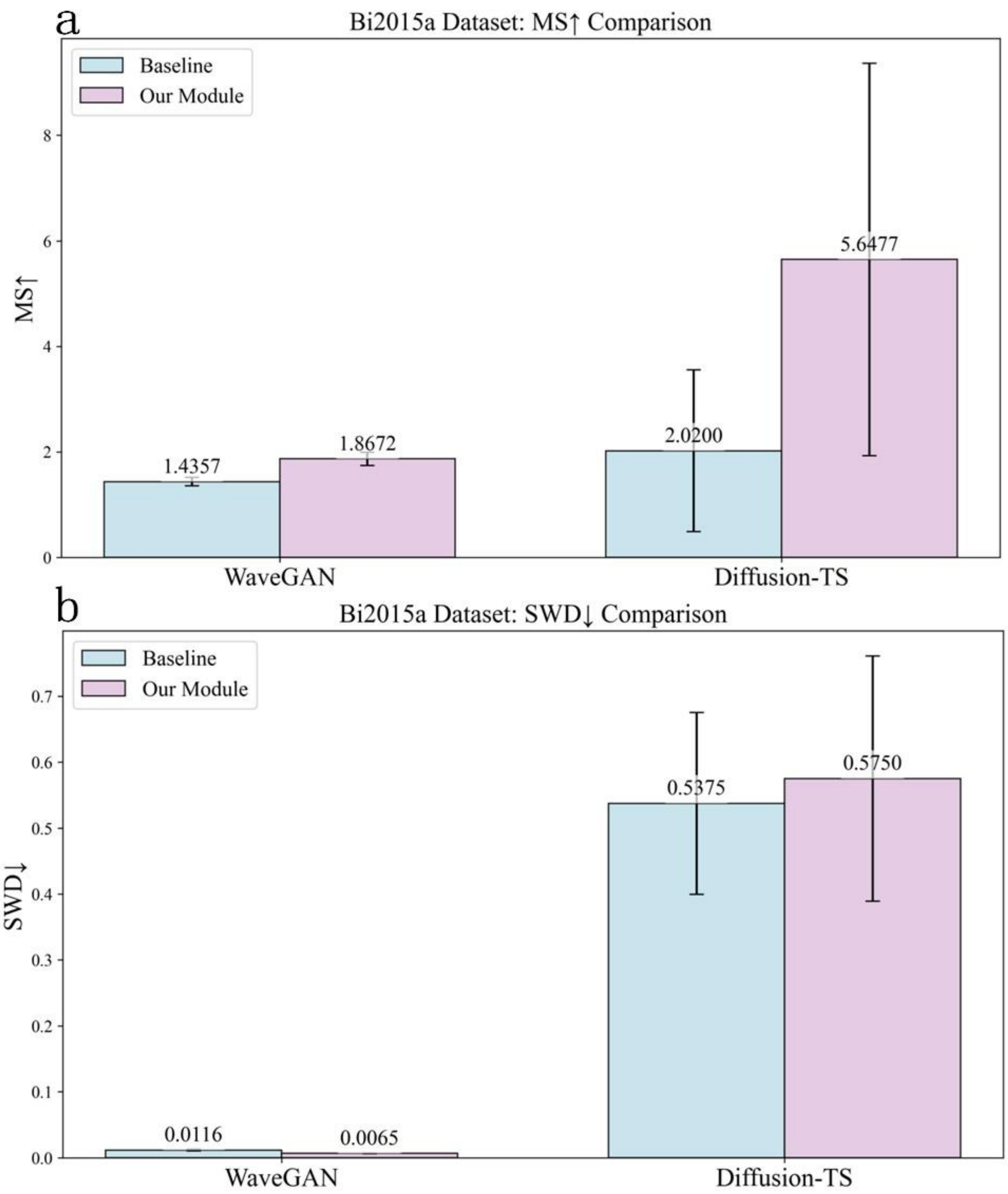

Figure S7: Quantitative evaluation of the MRM. (a). Comparison of MS scores. (b). Comparison of SWD scores. Data are represented as mean ± SD.

a

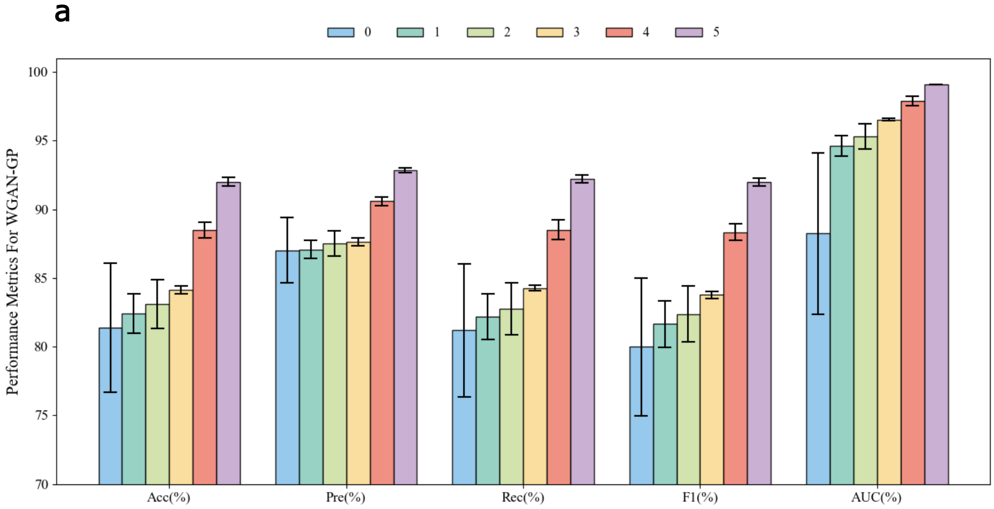

b

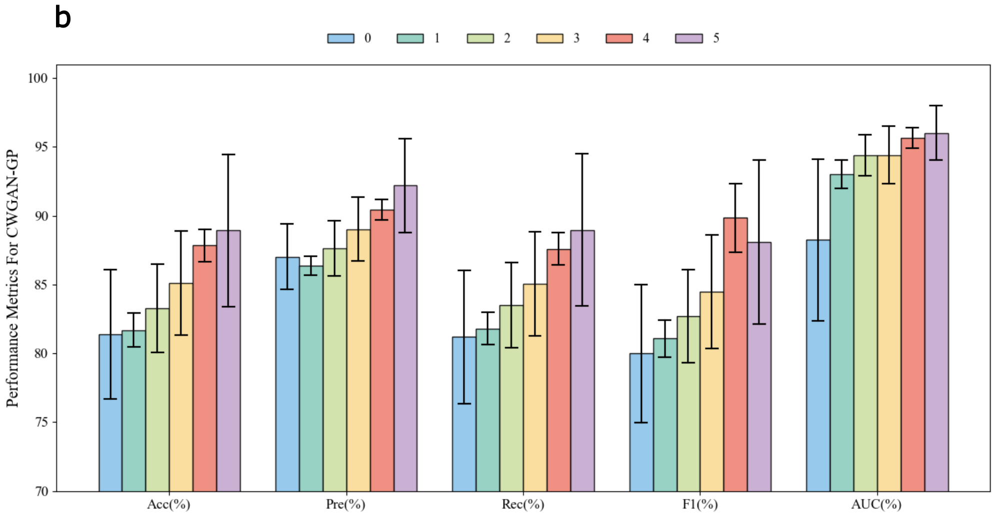

Figure S8: Two-class classification results based on different mixed ratios of five-channel EEG signals using EEGNet in the Bi2015a dataset. **(a)**.Classification performance of WGAN-GP. **(b)**.Classification performance of CWGAN-GP. Data are represented as mean ± SD.

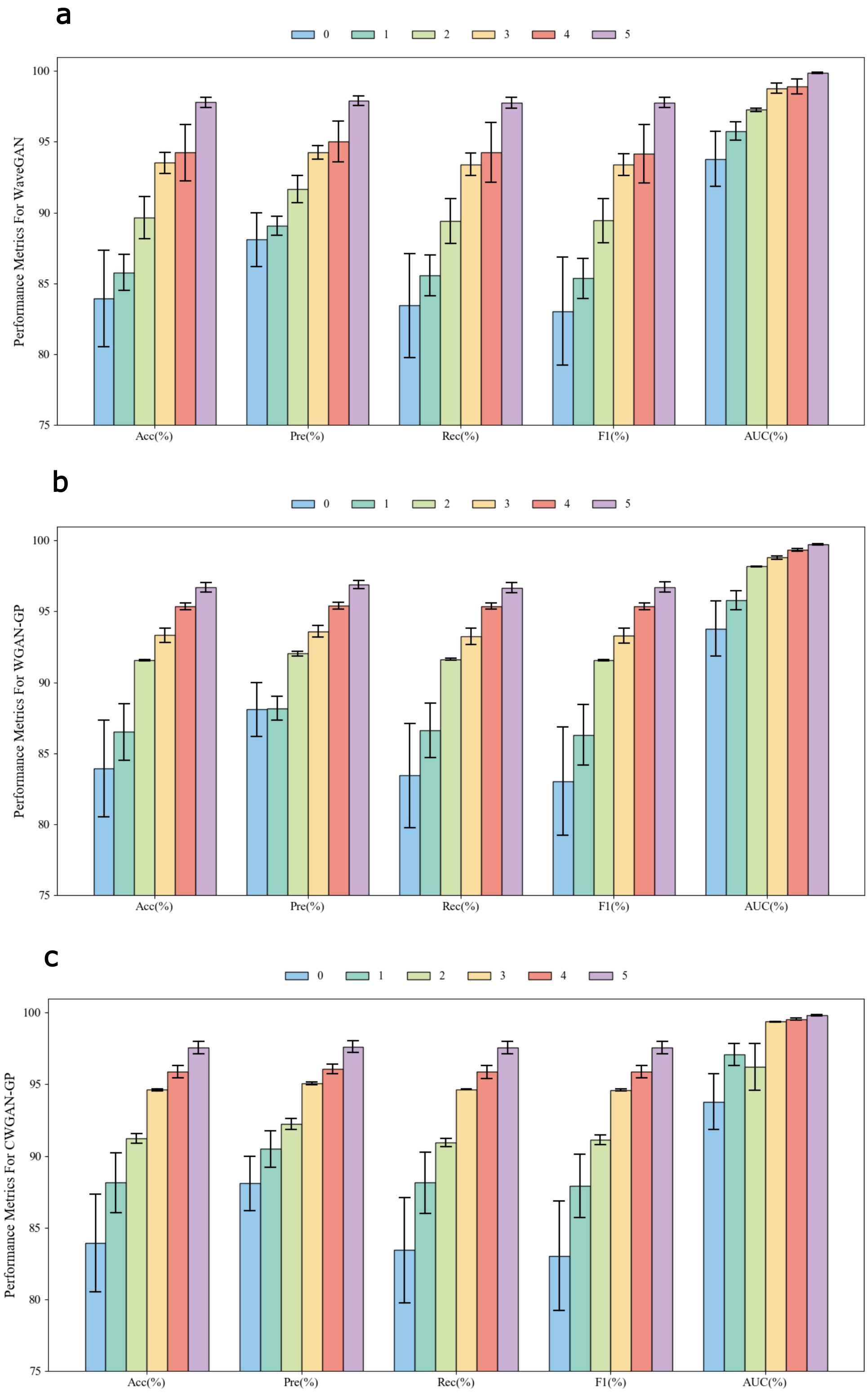

Figure S9: Two-class classification results based on different mixed ratios of five-channel EEG signals using EEGNet in the Bi2015a dataset. **(a)**.Classification performance of WaveGAN. **(b)**.Classification performance of WGAN-GP. **(c)**.Classification performance of CWGAN-GP. Data are represented as mean ± SD.

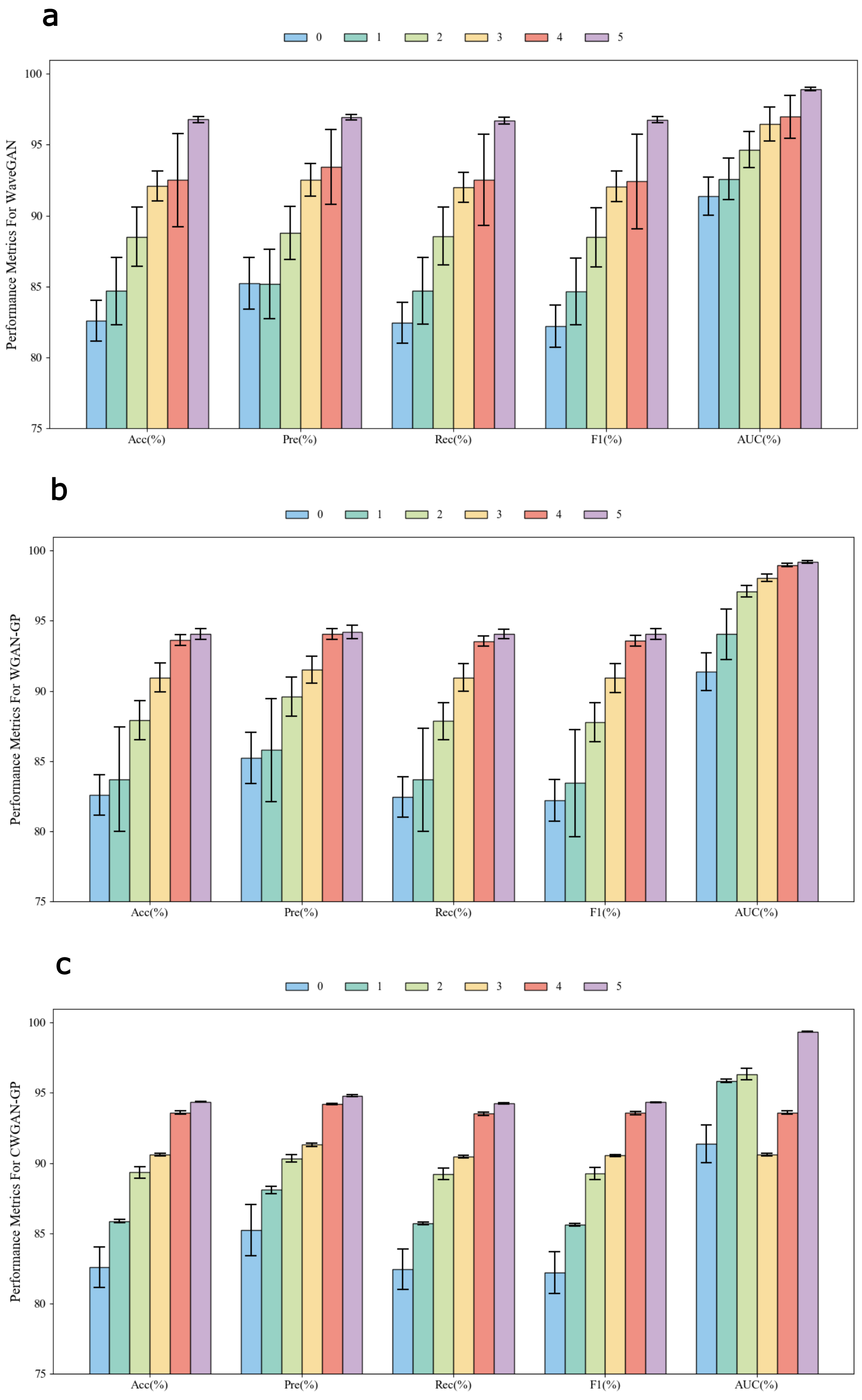

Figure S10: Two-class classification results based on different mixed ratios of five-channel EEG signals using EEGNet in the Bi2015a dataset. **(a)**.Classification performance of Wave-GAN. **(b)**.Classification performance of WGAN-GP. **(c)**.Classification performance of CWGAN-GP. Data are represented as mean ± SD.

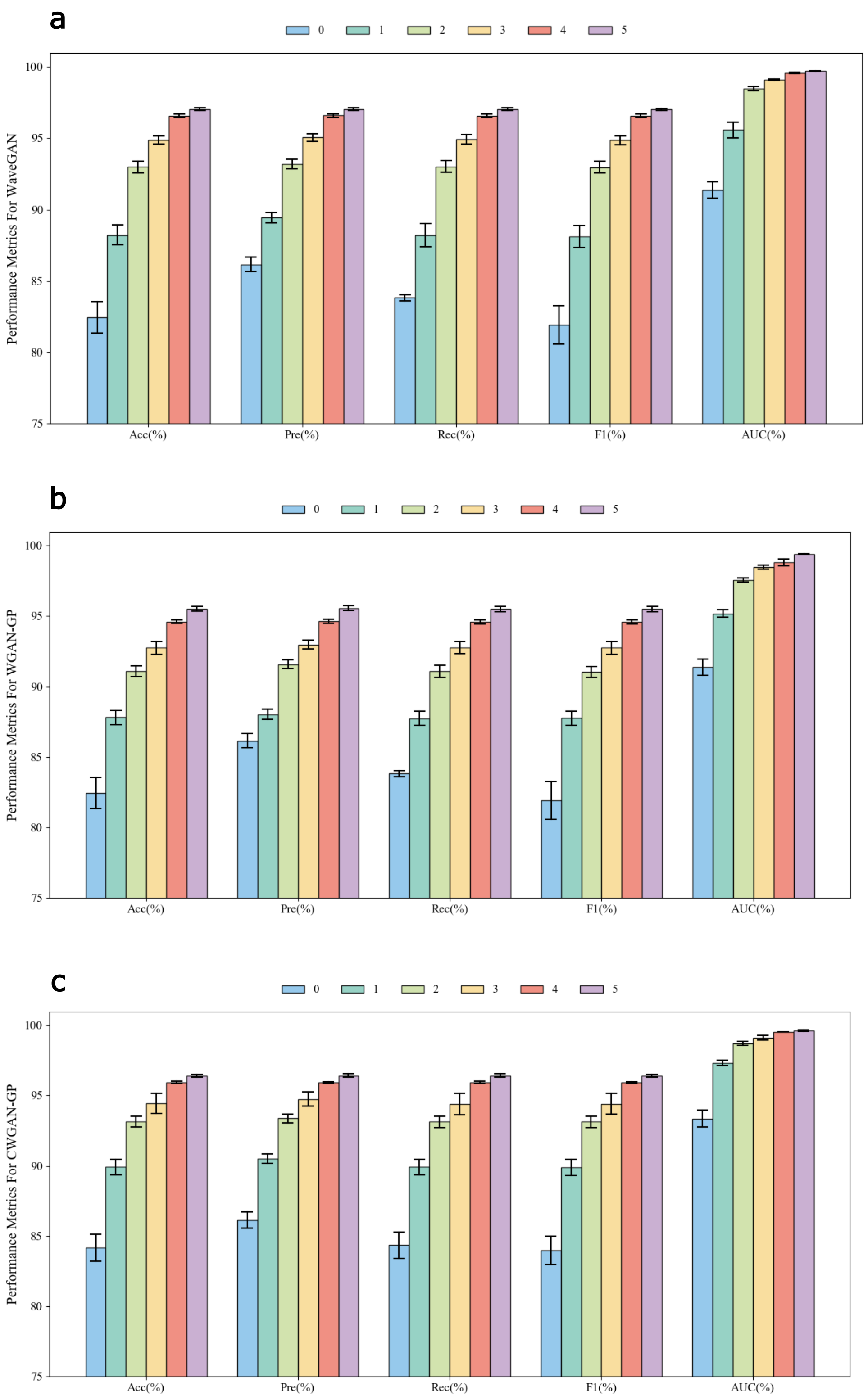

Figure S11: Two-class classification results based on different mixed ratios of five-channel EEG signals using EEGNet in the Bi2015a dataset. **(a)**.Classification performance of Wave-GAN. **(b)**.Classification performance of WGAN-GP. **(c)**.Classification performance of CWGAN-GP. Data are represented as mean ± SD.

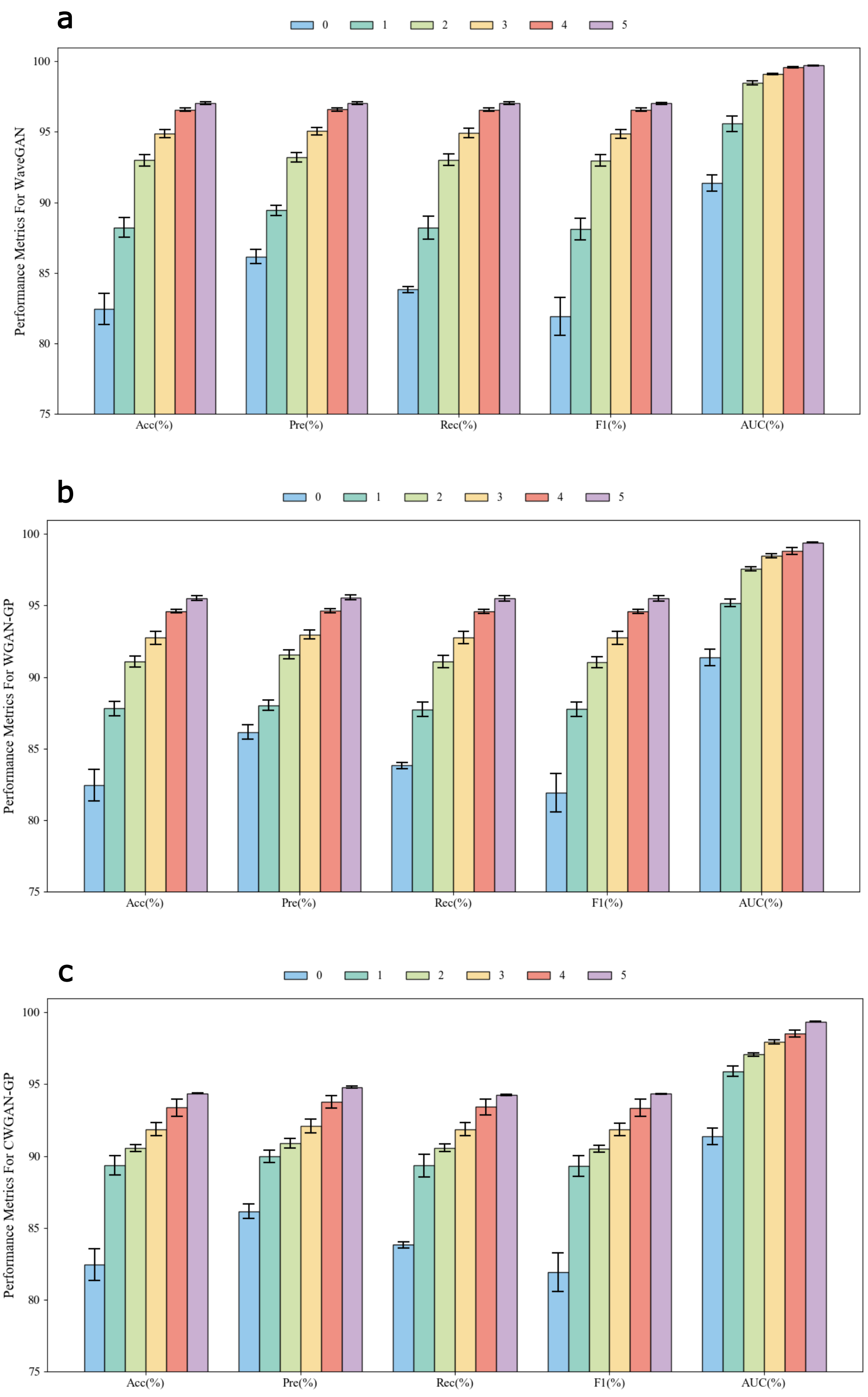

Figure S12: Two-class classification results based on different mixed ratios of five-channel EEG signals using EEGNet in the Bi2015a dataset. **(a)**.Classification performance of Wave-GAN. **(b)**.Classification performance of WGAN-GP. **(c)**.Classification performance of CWGAN-GP. Data are represented as mean ± SD.

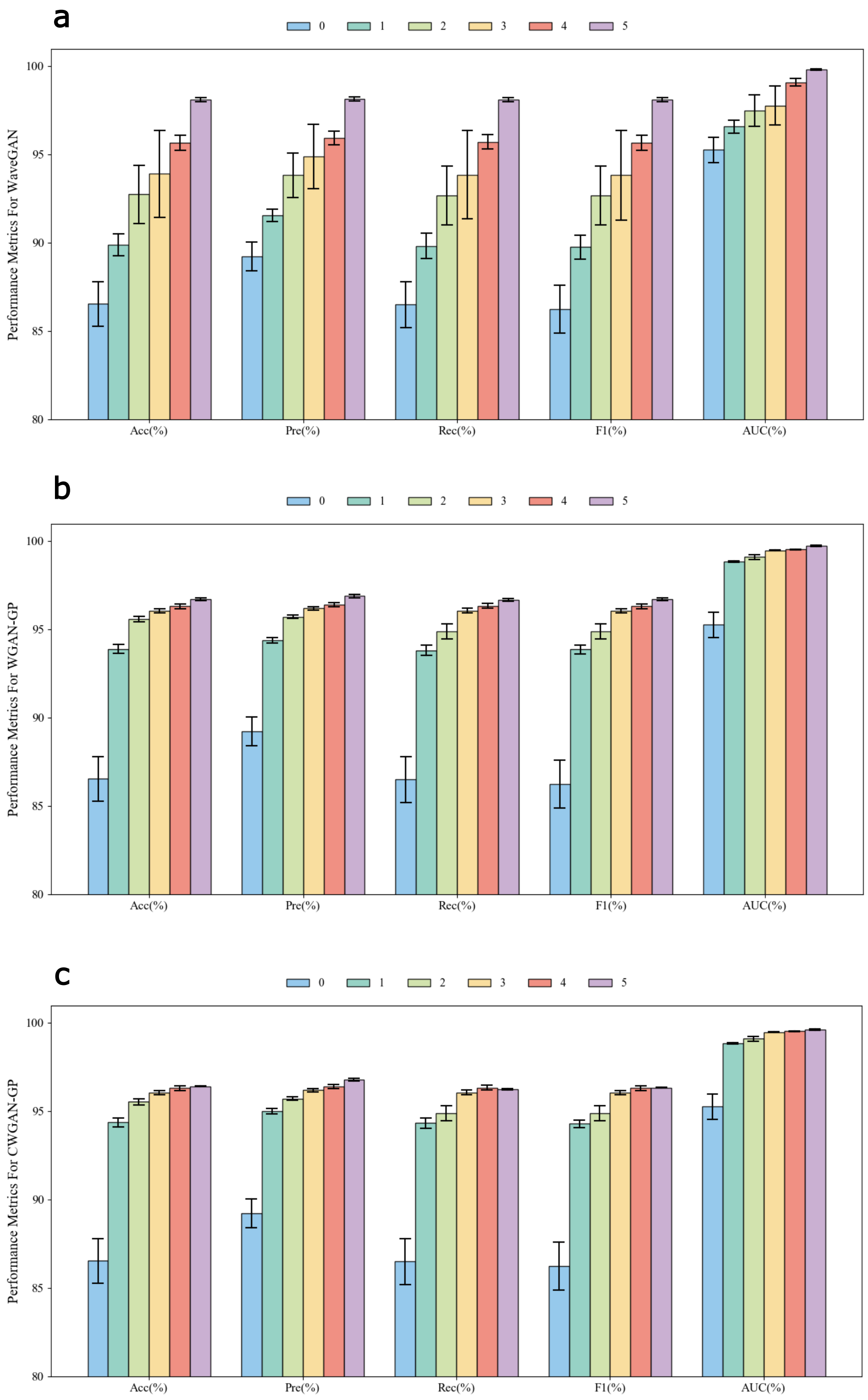

Figure S13: Two-class classification results based on different mixed ratios of five-channel EEG signals using EEGNet in the Bi2015a dataset. **(a)**.Classification performance of Wave-GAN. **(b)**.Classification performance of WGAN-GP. **(c)**.Classification performance of CWGAN-GP. Data are represented as mean ± SD.

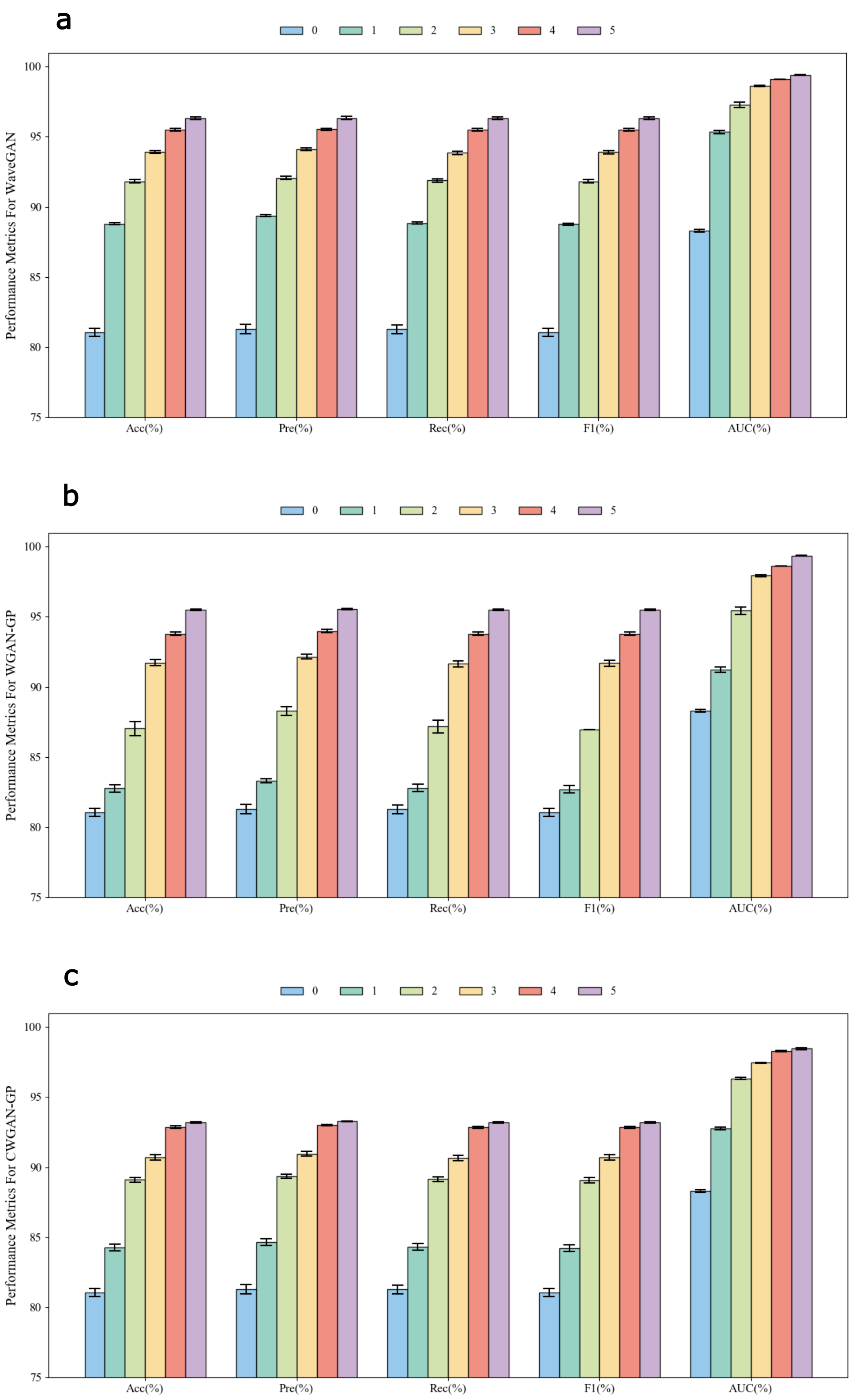

Figure S14: Two-class classification results based on different mixed ratios of five-channel EEG signals using EEGNet in the Bi2015a dataset. **(a)**.Classification performance of WaveGAN. **(b)**.Classification performance of WGAN-GP. **(c)**.Classification performance of CWGAN-GP. Data are represented as mean ± SD.

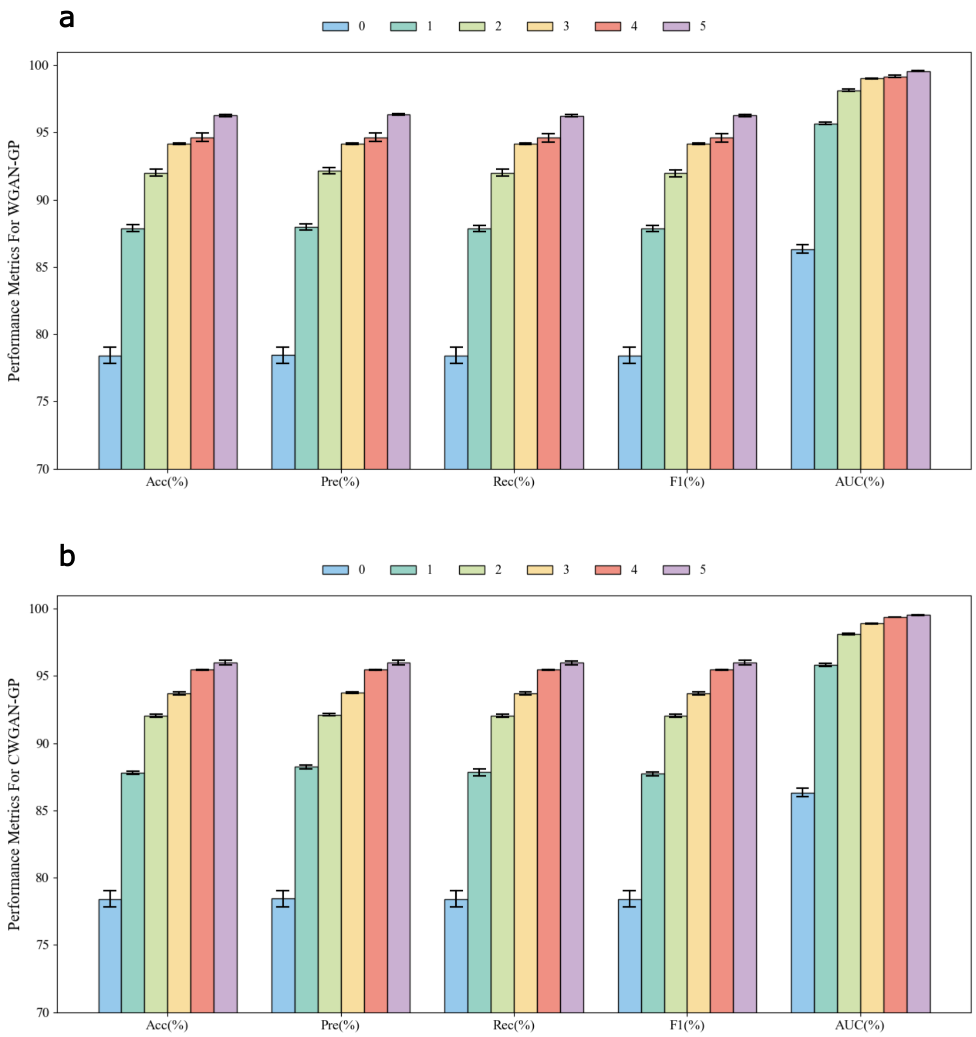

Figure S15: Two-class classification results based on different mixed ratios of five-channel EEG signals using EEGNet in the Bi2015a dataset. **(a)**.Classification performance of WGAN-GP. **(b)**.Classification performance of CWGAN-GP. Data are represented as mean ± SD.

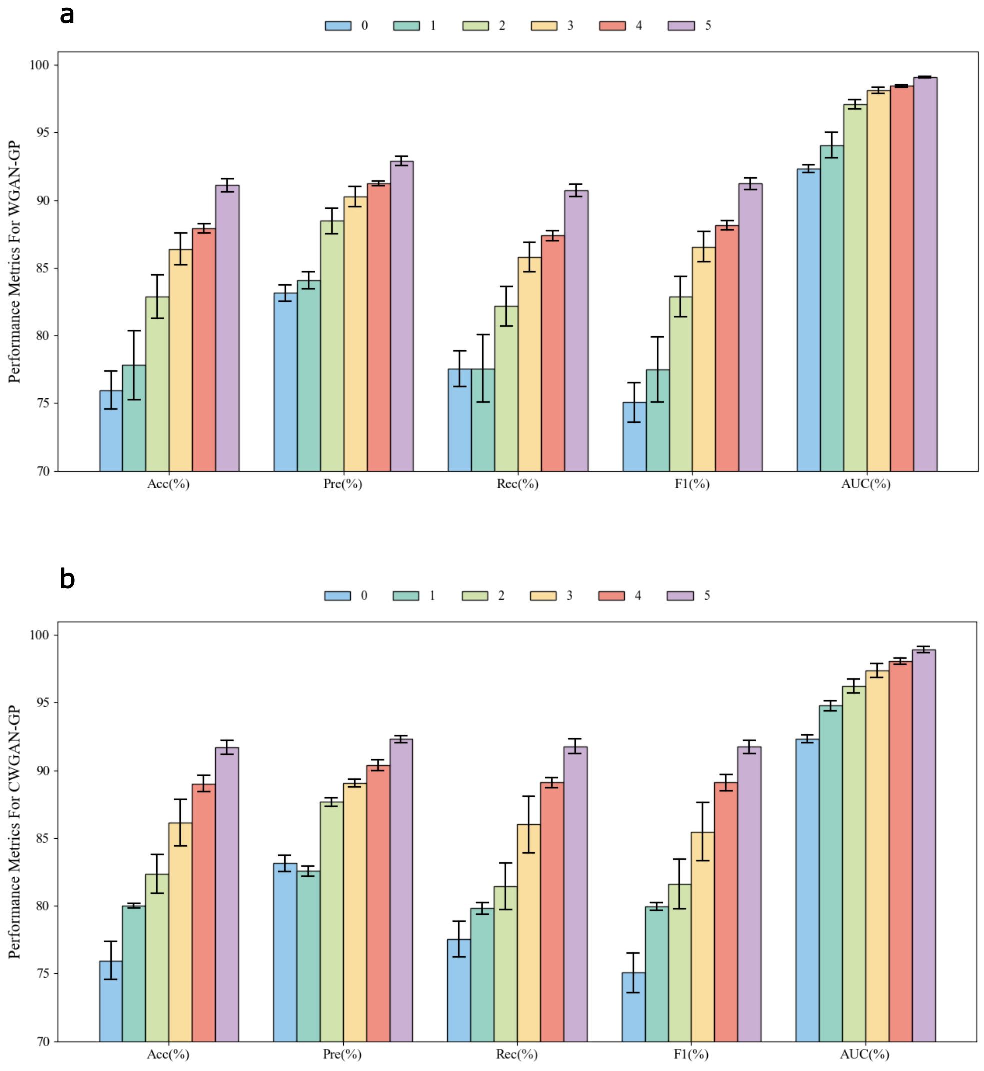

Figure S16: Two-class classification results based on different mixed ratios of five-channel EEG signals using EEGNet in the Bi2015a dataset. **(a)**.Classification performance of WGAN-GP. **(b)**.Classification performance of CWGAN-GP. Data are represented as mean ± SD.

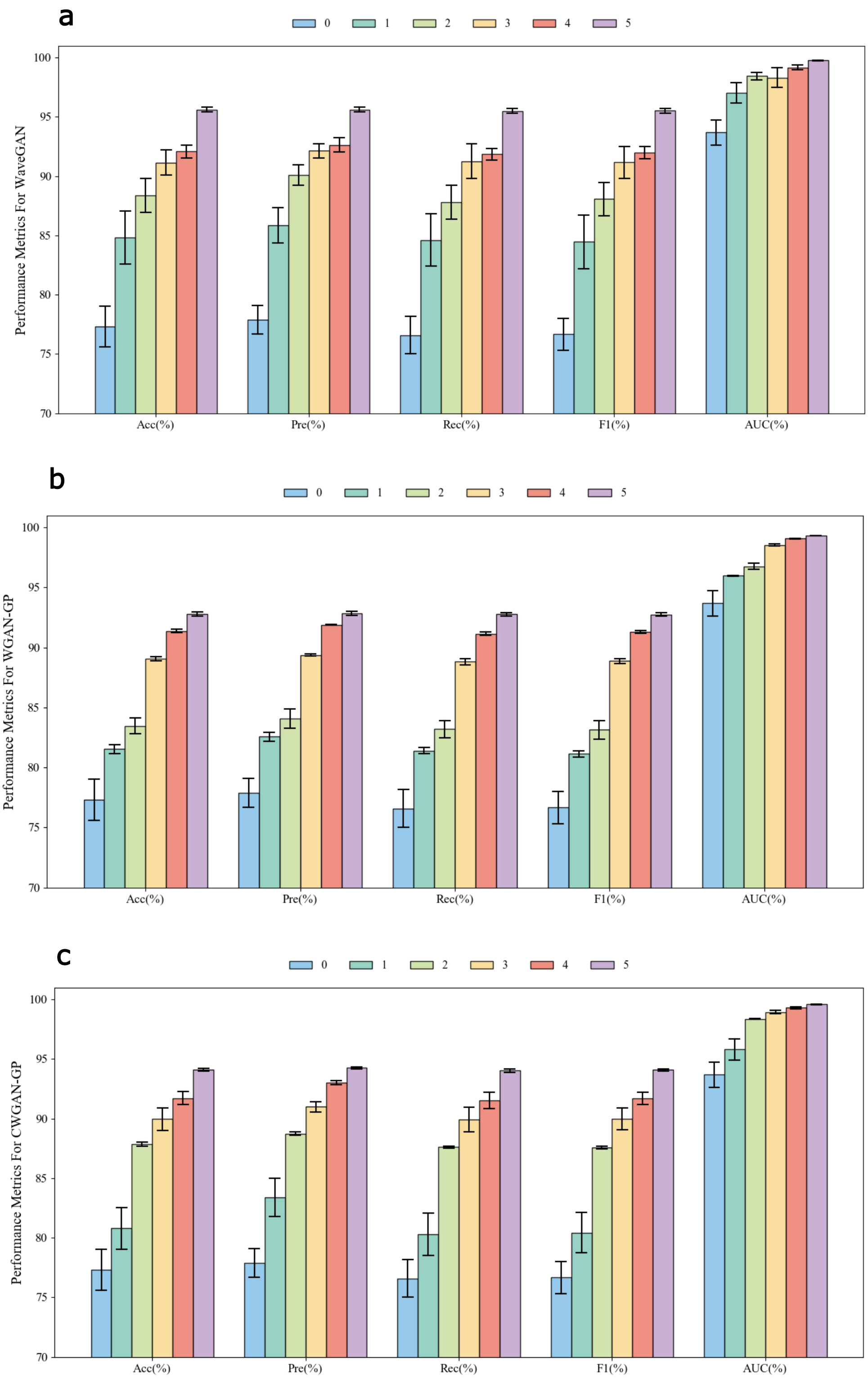

Figure S17: Two-class classification results based on different mixed ratios of five-channel EEG signals using EEGNet in the Bi2015a dataset. **(a)**.Classification performance of Wave-GAN. **(b)**.Classification performance of WGAN-GP. **(c)**.Classification performance of CWGAN-GP. Data are represented as mean ± SD.

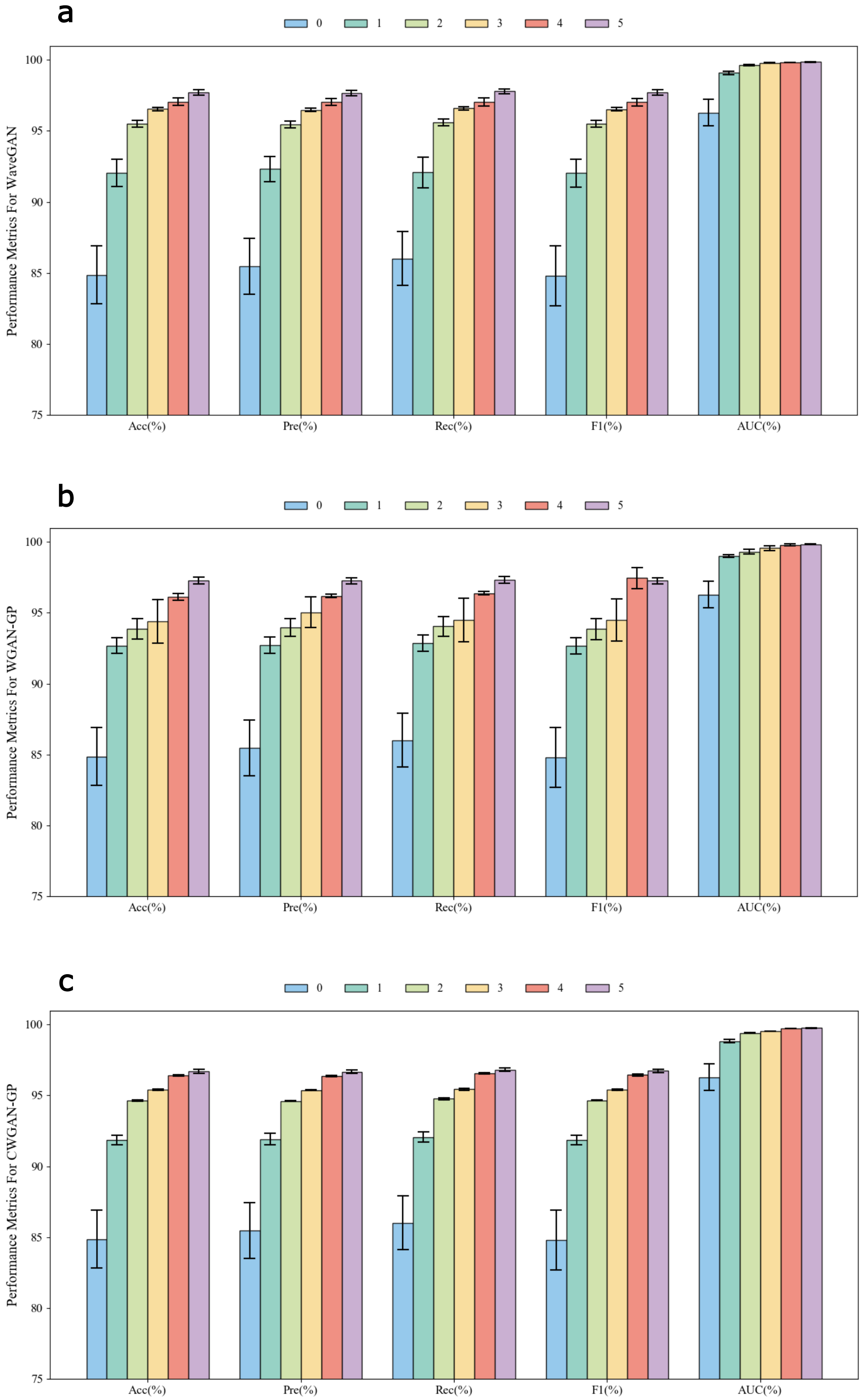

Figure S18: Two-class classification results based on different mixed ratios of five-channel EEG signals using EEGNet in the Bi2015a dataset. **(a)**.Classification performance of Wave-GAN. **(b)**.Classification performance of WGAN-GP. **(c)**.Classification performance of CWGAN-GP. Data are represented as mean ± SD.

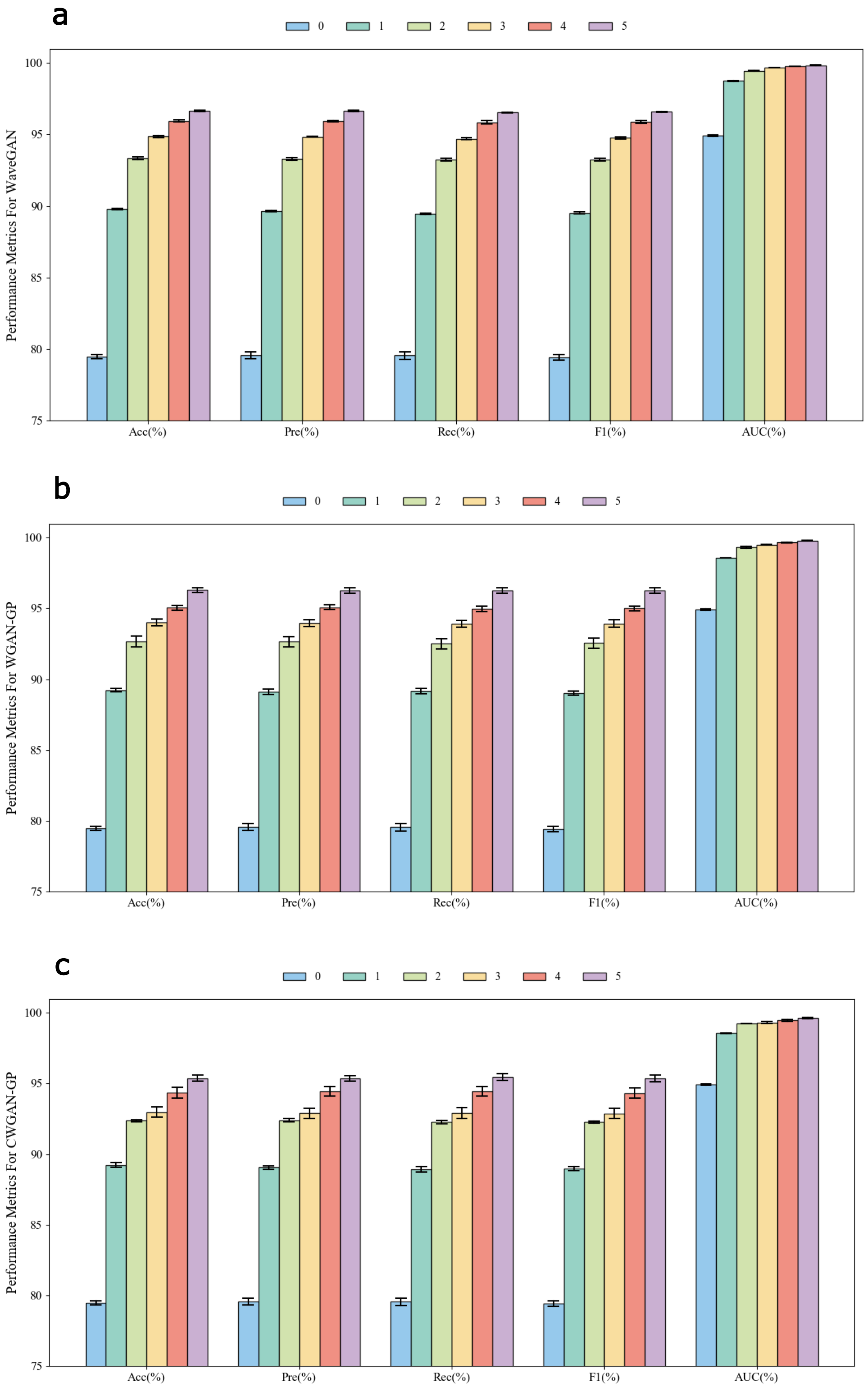

Figure S19: Two-class classification results based on different mixed ratios of five-channel EEG signals using EEGNet in the Bi2015a dataset. **(a)**.Classification performance of Wave-GAN. **(b)**.Classification performance of WGAN-GP. **(c)**.Classification performance of CWGAN-GP. Data are represented as mean ± SD.

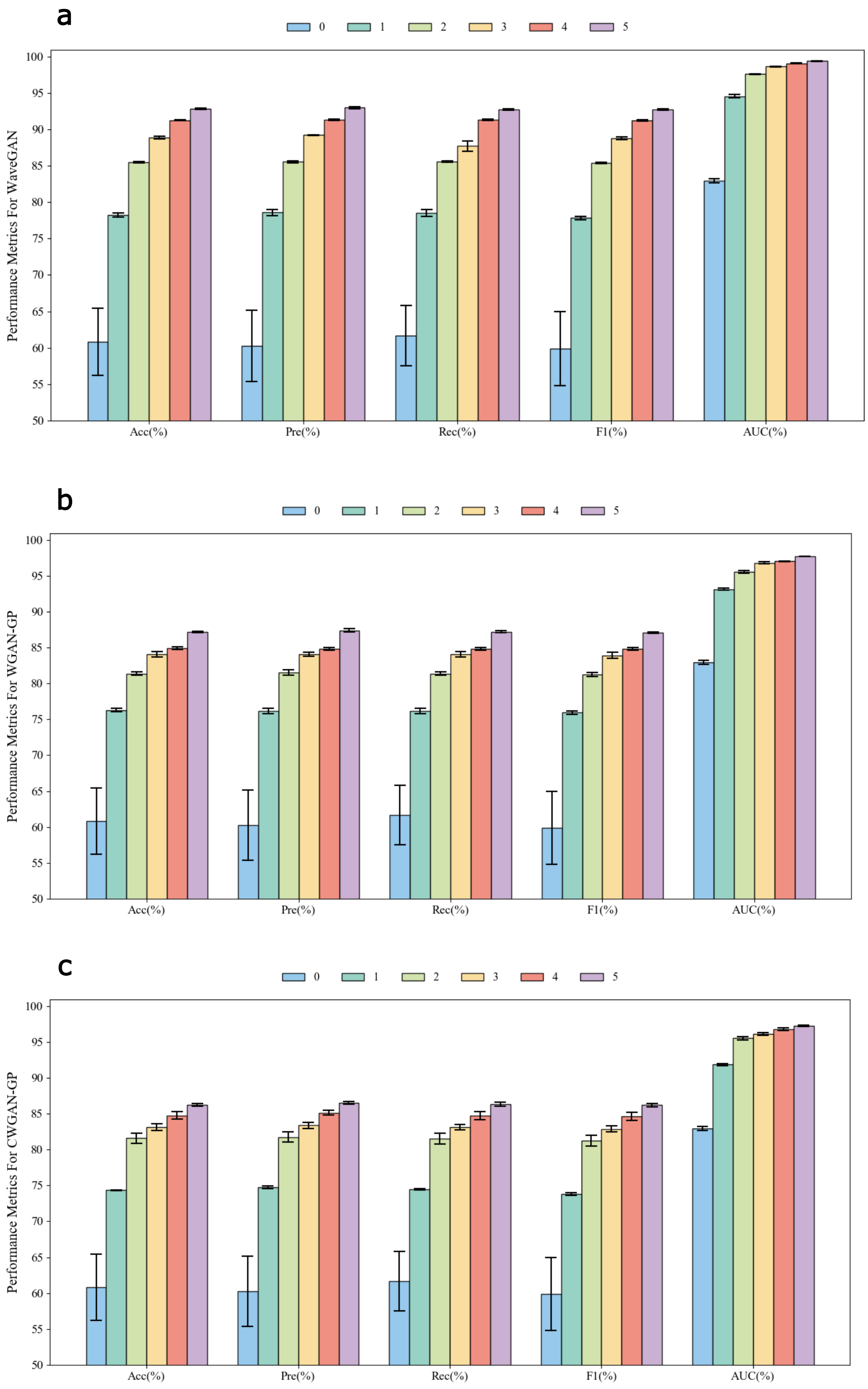

Figure S20: Two-class classification results based on different mixed ratios of five-channel EEG signals using EEGNet in the Bi2015a dataset. **(a)**.Classification performance of Wave-GAN. **(b)**.Classification performance of WGAN-GP. **(c)**.Classification performance of CWGAN-GP. Data are represented as mean ± SD.

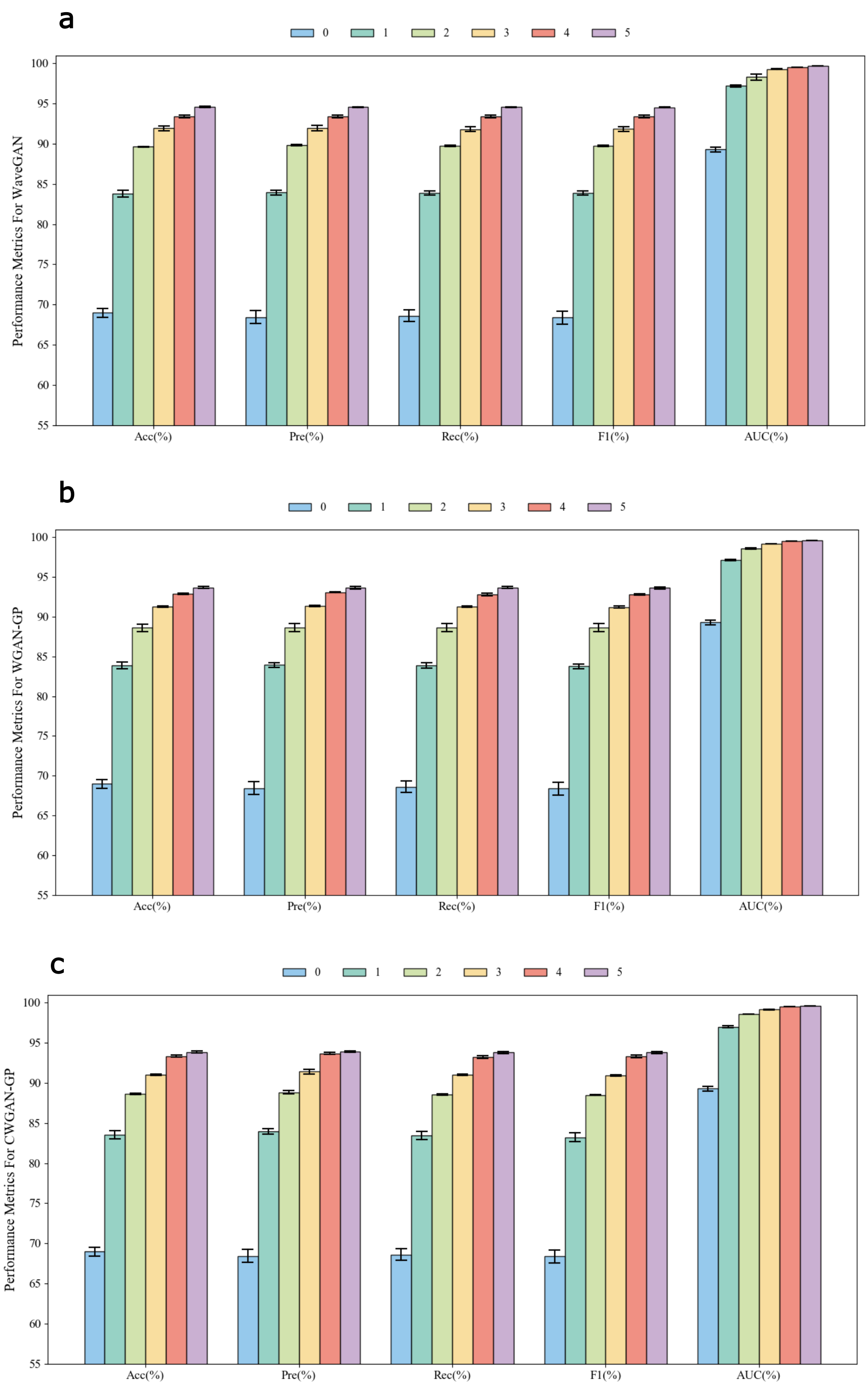

Figure S21: Two-class classification results based on different mixed ratios of five-channel EEG signals using EEGNet in the Bi2015a dataset. **(a)**.Classification performance of WaveGAN. **(b)**.Classification performance of WGAN-GP. **(c)**.Classification performance of CWGAN-GP. Data are represented as mean ± SD.

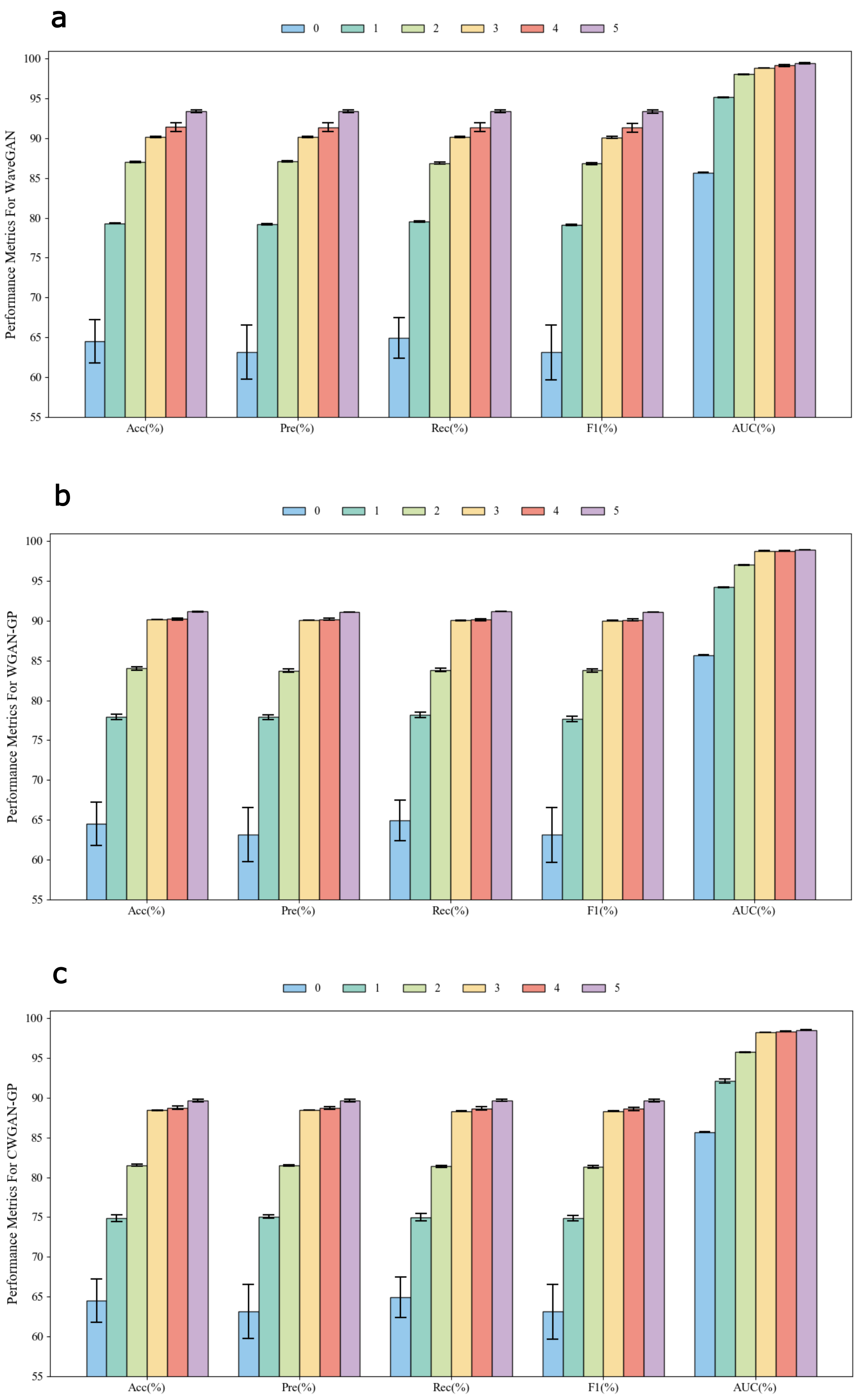

Figure S22: Two-class classification results based on different mixed ratios of five-channel EEG signals using EEGNet in the Bi2015a dataset. **(a)**.Classification performance of WaveGAN. **(b)**.Classification performance of WGAN-GP. **(c)**.Classification performance of CWGAN-GP. Data are represented as mean ± SD.

a

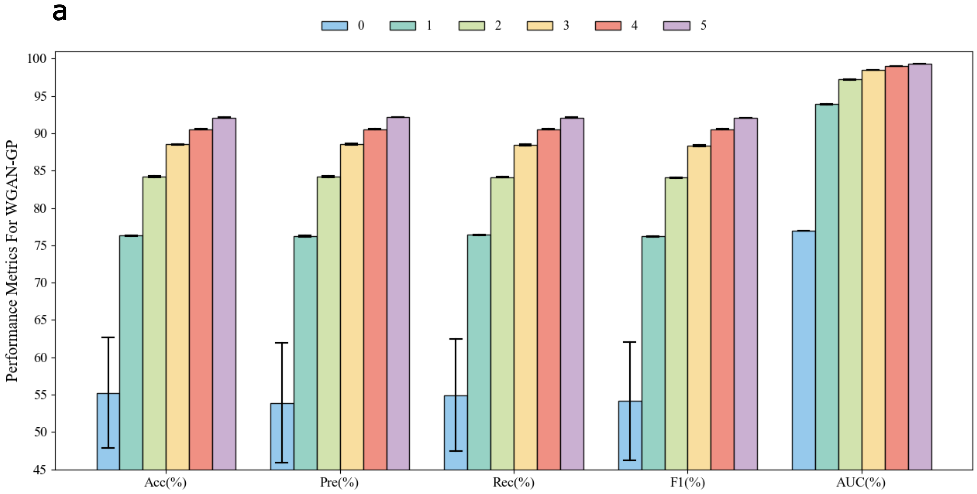

b

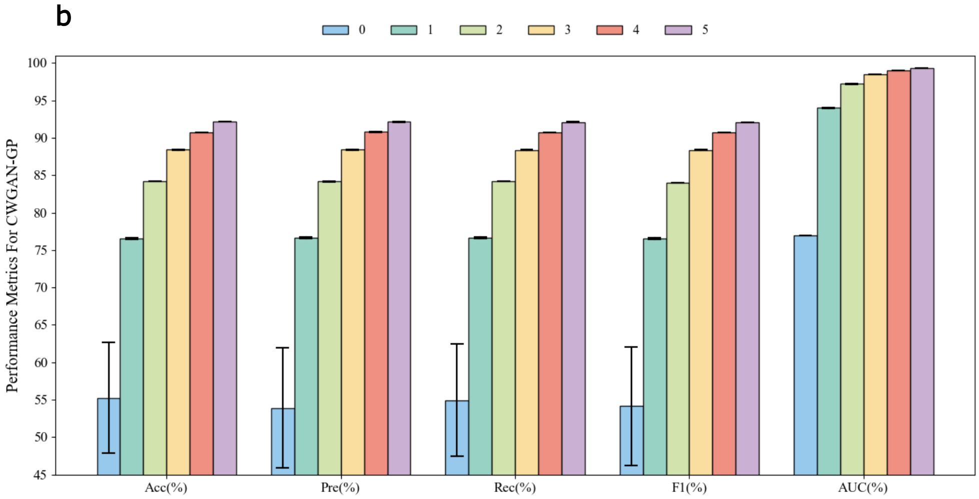

Figure S23: Two-class classification results based on different mixed ratios of five-channel EEG signals using EEGNet in the Bi2015a dataset. **(a)**.Classification performance of WGAN-GP. **(b)**.Classification performance of CWGAN-GP. Data are represented as mean ± SD.
